# Multi‐Omics Integration Identifies a CDH3‐Associated Malignant Epithelial State and Immunosuppressive Niche to Predict Prognosis in Thymic Epithelial Tumors

**DOI:** 10.1002/advs.77153

**Published:** 2026-08-15

**Authors:** Yuntao Feng, Jingyu Chen, Lang Xia, Tao Wang, Yuzhou Wang, Lei Zhang, Guofang Zhao, Long Xu, Juemin Yu, Yunlang She, Junqi Wu, Yue Zhao, Chang Chen, Deping Zhao

**Affiliations:** ^1^ Department of Thoracic Surgery School of Medicine Shanghai Pulmonary Hospital Tongji University Shanghai China; ^2^ Department of Thoracic Surgery Ningbo Second Hospital Ningbo China

**Keywords:** CDH3, computational pathology, M2 macrophages, multi‐omics integration, thymic epithelial tumors

## Abstract

Thymic epithelial tumors (TETs) are rare and heterogeneous malignancies whose aggressive epithelial states and microenvironmental organization remain poorly defined. Here, we integrated single‐cell RNA sequencing, spatial transcriptomics, multiplex immunofluorescence, bulk transcriptomics, functional assays, xenograft validation, and computational pathology to characterize malignant epithelial heterogeneity in TETs. We identified a CDH3‐associated malignant epithelial state located at the origin of malignant‐state trajectories and enriched for stem‐like and EMT‐related features. Spatial transcriptomics and multiplex immunofluorescence showed that CDH3+ tumor cells preferentially localized within an M2 macrophage‐rich immunosuppressive niche, while cell–cell communication analyses nominated CCN2–LRP1 as a candidate epithelial–myeloid crosstalk axis. A 68‐gene CDH3‐associated signature stratified TCGA‐THYM into biologically distinct subgroups with differences in survival, histology, genomic instability, and immune contexture. Patient‐derived thymic carcinoma organoids showed elevated CDH3 expression, and CDH3 silencing suppressed thymic carcinoma cell proliferation, migration, invasion, EMT/PI3K–Akt‐related signaling, and macrophage‐associated crosstalk. Candidate inhibitors showed antitumor activity in xenograft models. We also established a deep learning pathology model that captured CDH3‐associated morphology from routine H&E slides and predicted patient outcome. Together, these findings define CDH3 as a biomarker and therapeutic target linking malignant epithelial plasticity to immunosuppressive niche formation and adverse clinical behavior in TETs.

## Graphical abstract

Single‐cell, spatial, molecular, and pathology analyses identify a CDH3‐associated malignant epithelial state in thymic epithelial tumors. This state links stem‐like and EMT programs to M2 macrophage–rich immunosuppressive niches, genomic instability, poor survival, and drug vulnerability. A pathology‐based model further captures CDH3‐high morphology and predicts patient risk from routine H&E slides.

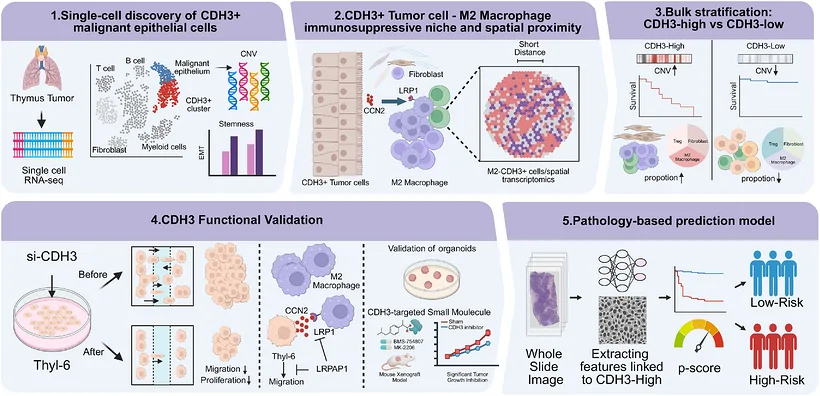

## 1 Introduction

Thymic epithelial tumors (TETs) are rare yet highly heterogeneous anterior mediastinal neoplasms, with an annual incidence of approximately 0.13–0.32 per 100 000 population [1]. TETs mainly comprise two pathological subtypes: thymomas and thymic carcinoma. According to the latest 2021 WHO classification, thymomas are further subdivided into five histological types: A, AB, B1, B2, and B3 [2]. All major subtypes of TET have inherent invasive potential [3], with B2/B3 thymomas and thymic carcinoma exhibiting particularly aggressive behavior, frequently invading adjacent mediastinal structures at an early stage and being associated with high rates of recurrence and metastasis [4], which collectively result in an overall 5‐year survival rate that remains persistently below 50% [5, 6]. Further studies are urgently needed to elucidate the tumorigenic mechanisms of TETs and to identify potential therapeutic targets.

Recent advances in single‐cell and spatial transcriptomic profiling have made it possible to resolve tumor ecosystems with much greater precision, linking malignant cell states to stromal and immune context within intact tissues [7, 8]. These approaches are especially relevant for TETs, whose biology is shaped by both epithelial heterogeneity and an unusually complex microenvironment. However, the aggressive epithelial states that drive progression in TETs remain poorly defined, and it is still unclear how such states are spatially organized or coupled to local immune‐stromal niches. As a result, the relationships among epithelial plasticity, tissue architecture, and clinical behavior in TETs remain incompletely understood.

Among the processes most closely associated with tumor progression, epithelial‐mesenchymal transition (EMT) and related forms of epithelial plasticity are linked to invasion, stem‐like features, immune evasion, and treatment resistance across solid tumors [9, 10, 11, 12, 13]. At the same time, histopathology remains the clinical foundation for TET diagnosis, raising the possibility that high‐risk epithelial programs may be reflected in recognizable morphologic patterns. Integrating transcriptomic, spatial, and histopathologic information therefore offers an opportunity to connect malignant cell states with tissue organization and to translate biologic heterogeneity into clinically accessible features [14].

Here, we integrated bulk, single‐cell, spatial, and histopathologic data to interrogate malignant epithelial heterogeneity in TETs. We sought to identify epithelial programs associated with aggressive disease, determine how these programs are embedded within local immune‐stromal microenvironments, and test whether they can be captured as clinically relevant molecular and morphologic phenotypes. Through this framework, we aimed to link epithelial plasticity in TETs to disease progression and therapeutic vulnerability.

## 2 Results

### 2.1 Integrated Single‐Cell Profiling Maps the TET Ecosystem

The mechanisms driving malignant initiation and progression of thymic epithelial tumors remain poorly defined, and tumor cell composition and microenvironmental interactions are incompletely characterized, hindering clinical diagnostic and therapeutic optimization. We therefore integrated and analyzed publicly available single‐cell transcriptomic data to dissect malignancy‐associated cellular features and tumor microenvironment interactions.

Following quality control and batch effect correction of the raw data using the Seurat package, a total of 51 463 cells were retained for downstream analysis. Based on the expression patterns of well‐established marker genes, we identified six major cell types, including T cells, B cells, epithelial cells, endothelial cells, myeloid cells, and stromal cells (Figure S1A,B). The distribution of cell type–specific marker gene expression was consistent with canonical lineage identities—for example, EPCAM was highly enriched in epithelial cells, PECAM1 specifically marked endothelial cells, MS4A1/CD79A characterized B cells, TRBC1 marked T cells, LYZ/CD14 identified myeloid cells, and RGS5/PDGFRB were predominantly expressed in stromal cells. Cell‐type proportions varied across individual samples, reflecting patient‐to‐patient variability in the cellular composition of the TET microenvironment (Figure S1C). Consistently, feature‐density visualization further illustrated the spatially distinct enrichment patterns of representative markers across the global cell atlas (Figure S1D). To further characterize the epithelial compartment, epithelial cells were re‐clustered and subjected to copyKAT analysis to infer large‐scale chromosomal copy number variations, and epithelial cells exhibiting widespread aneuploidy were annotated as tumor cells (Figure S1E–G).

### 2.2 A CDH3^+^ Malignant Epithelial Subpopulation Exhibits Stem‐Like and Pro‐Invasive Features in TETs

To further characterize intratumoral heterogeneity, we extracted all malignant epithelial cells defined by CopyKAT and performed unsupervised re‐clustering, which resolved five distinct cancer cell subpopulations (C0_PDK4, C1_SLPI, C2_CDH3, C3_GMFG, and C4_RGS1), each characterized by subtype‐specific marker patterns (Figure 1A,B). To link intratumoral heterogeneity to clinical outcomes, we integrated our single‐cell malignant compartment with TCGA bulk transcriptomes and survival information using Scissor, which identifies phenotype‐associated single‐cell subpopulations by leveraging bulk‐omics phenotypes (Figure 1C,D). Notably, C1_SLPI and C2_CDH3 were enriched for Scissor+ cells (and accounted for a substantial fraction of malignant cells), suggesting that these states are preferentially associated with unfavorable survival‐related bulk signatures in TETs.

**FIGURE 1:**
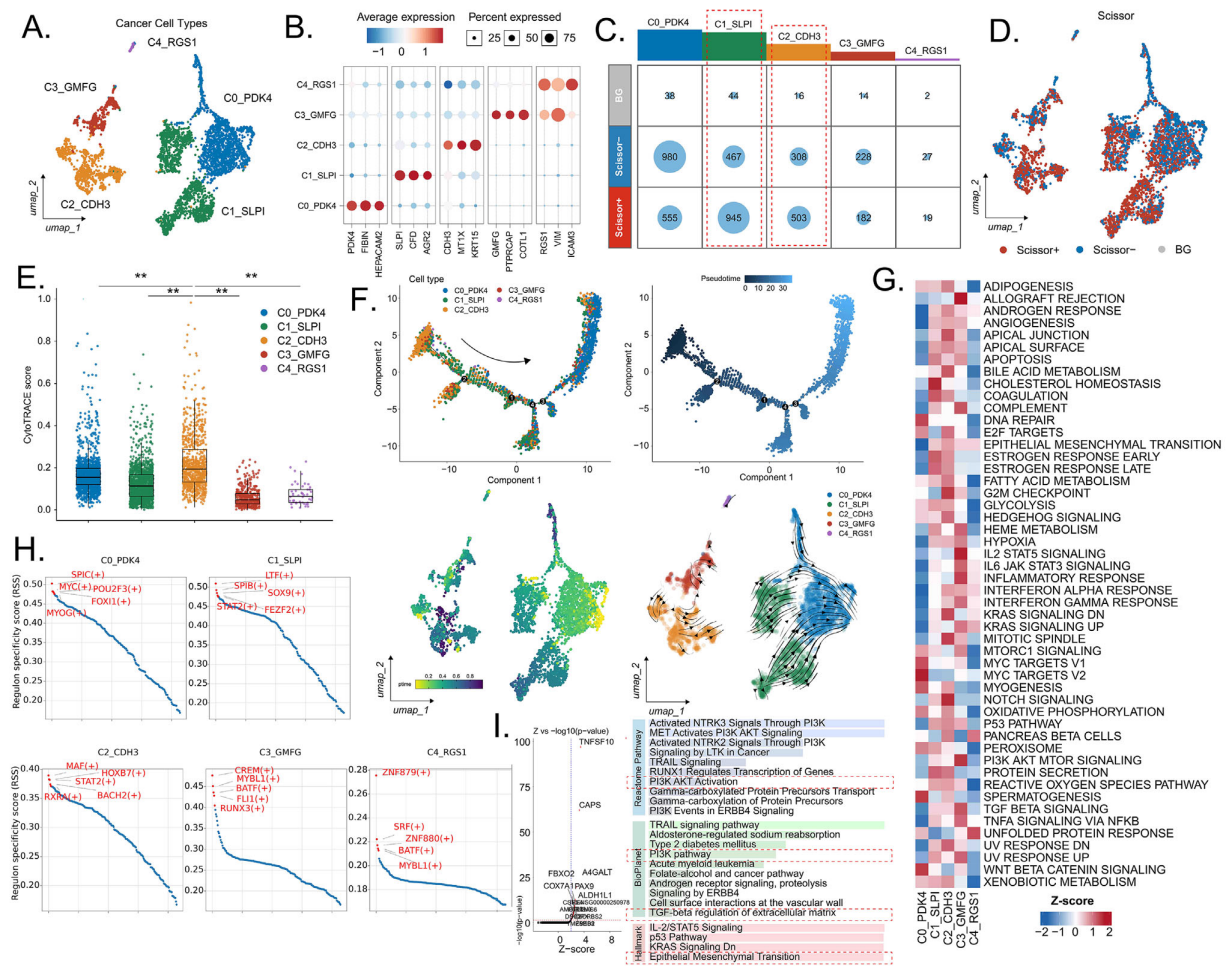
A CDH3‐associated malignant epithelial subpopulation exhibits stem‐like and pro‐invasive features in TETs. (A) UMAP visualization of CopyKAT‐defined malignant epithelial cells. (B) Dot plot showing the expression of representative marker genes across the five malignant epithelial subpopulations. (C) Distribution of Scissor‐assigned cells across malignant epithelial subpopulations. (D) UMAP plot showing the distribution of Scissor+, Scissor−, and background cells across malignant epithelial states. (E) Comparison of CytoTRACE scores across malignant epithelial subpopulations. (F) Trajectory inference analyses using Monocle2 and scTour. (G) Heatmap of Hallmark pathway activity scores across malignant epithelial subpopulations. (H) SCENIC regulon specificity analysis. (I) Functional enrichment analysis of perturbed genes predicted by in silico CDH3 knockout in C2_CDH3 cells using scTenifoldKnk. ^**^ *p* < 0.01 vs. the C2_CDH3.

We then evaluated differentiation potential within malignant cells using CytoTRACE, which infers relative developmental potential/stemness from scRNA‐seq. Among the five malignant subpopulations, C2_CDH3 consistently displayed the highest CytoTRACE scores (Figure 1E), indicating the strongest stem‐like features. To further interrogate malignant‐state dynamics, we performed trajectory inference using both Monocle2 and the deep‐learning–based method scTour. Both approaches identified C2_CDH3 as the starting point of the inferred trajectory, suggesting that it represents an early, stem‐like malignant state (Figure 1F).

To characterize pathway‐level programs across malignant subpopulations, we scored activity of the 50 MSigDB Hallmark gene sets. C2_CDH3 showed significant enrichment of multiple signaling pathways associated with malignant progression, including EMT and NOTCH, suggesting enhanced cellular plasticity and pro‐migratory potential (Figure 1G). SCENIC analysis further revealed marked state‐specific differences in transcriptional regulatory networks among malignant states, with C2_CDH3 displaying a distinct and strongly activated EMT‐related regulatory program (Figure 1H).

Finally, to probe potential functional dependencies, we performed scTenifoldKnk virtual perturbation of CDH3 within C2_CDH3 cells. Predicted downstream impacts were enriched in cancer‐relevant programs, including PI3K‐related signaling, EMT, and TGF‐β signaling, among others (Figure 1I), supporting CDH3 as a candidate regulator of the stem‐like and pro‐invasive malignant program.

### 2.3 Intercellular Communication Analysis Maps the TET Microenvironment

To refine the organization of the immune and stromal compartments, we further subclustered the major non‐malignant lineages into functionally distinct subsets, including naïve, effector, tissue‐resident and regulatory T‐cell states, antibody‐secreting, germinal‐center‐like and naïve B cells, myeloid subsets such as dendritic cells, monocytes, M1 and M2 macrophages, and stromal populations including fibroblasts and vascular smooth muscle cells. The integrated UMAP of all refined cell subsets and the corresponding lineage‐specific UMAPs illustrated clear separation of these populations across the TET ecosystem (Figure 2A and Figure S2A). Their identities were further supported by canonical marker expression patterns (Figure S2B). The relative abundance of refined immune and stromal subsets differed across samples (Figure S2C).

**FIGURE 2:**
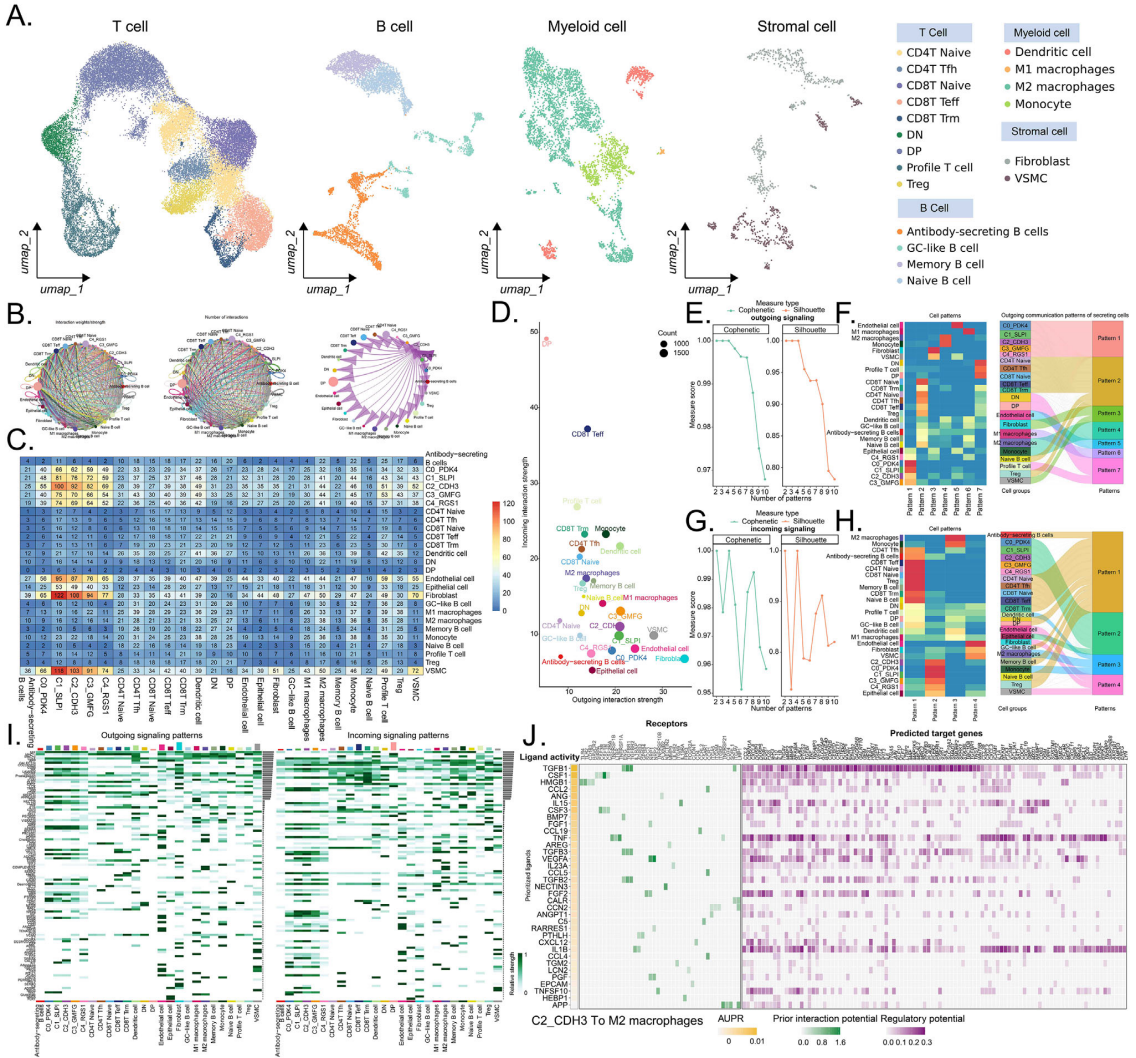
Intercellular communication analysis maps the immune–stromal ecosystem in TETs. (A) Lineage‐specific UMAP visualizations of refined non‐malignant cell subsets in the TET microenvironment. (B) Global CellChat network visualizations showing the overall intercellular communication landscape. (C) Heatmap of interaction counts among all annotated malignant, immune, and stromal cell subsets. (D) Two‐dimensional signaling role analysis summarizing outgoing and incoming interaction strengths for each cell subset. (E) Determination of the optimal number of outgoing communication patterns based on cophenetic and silhouette scores. (F) Outgoing communication pattern analysis. (G) Determination of the optimal number of incoming communication patterns based on cophenetic and silhouette scores.(H) Incoming communication pattern analysis. (I) Heatmaps summarizing outgoing and incoming signaling pathway activities across all annotated cell subsets. (J) NicheNet ligand–receptor–target inference for signaling from CDH3^+^ malignant cells to M2 macrophages.

Global CellChat network visualization revealed extensive inter‐compartment signaling across the ecosystem, and highlighted C2_CDH3 malignant cells as a prominent signaling source interacting with multiple immune and stromal populations (Figure 2B). The interaction count heatmap further illustrated the overall connectivity landscape among all annotated cell subsets, showing broad communication links spanning malignant epithelial, myeloid, lymphoid, and stromal compartments (Figure 2C). In line with this, signaling role analysis in two‐dimensional space showed C2_CDH3 among the cell types with strong outgoing communication strength, whereas M2 macrophages, fibroblasts and antibody‐secreting B cells ranked among the dominant signal recipients (Figure 2D). Communication pattern analysis further organized outgoing signals into seven major patterns (Figure 2E,F), indicating that distinct sender cell programs coordinately contribute to different signaling modules across the ecosystem. In parallel, incoming signals could be grouped into four major patterns (Figure 2G,H), with M2 macrophages emerging as a principal contributor to the Pattern 3 receiver program, consistent with their prominent involvement in the malignant niche.

At the pathway level, signaling‐role heatmaps summarized functional communication programs across cell subsets and revealed coherent outgoing and incoming signaling modules within the aggregated communication network (Figure 2I). To further resolve the downstream effects of CDH3^+^ malignant epithelial signaling on individual microenvironmental populations, we performed NicheNet analysis across multiple recipient cell subsets, including fibroblasts, M1 macrophages, CD4^+^ T cells, CD8^+^ T cells, naïve B cells, and Treg cells (Figure S2D–I), indicating broad regulatory interactions between malignant epithelial cells and both stromal and immune compartments. Notably, when focusing on the interaction between CDH3^+^ malignant epithelial cells and Pattern 3–associated M2 macrophages, NicheNet‐based ligand–receptor–target inference prioritized the CCN2–LRP1 axis, accompanied by coordinated changes in a set of EMT‐associated downstream target genes (Figure 2J).

### 2.4 Spatial Transcriptomics Reveals a CDH3^+^ Malignant Niche in TETs

To investigate how malignant epithelial states are spatially organized within thymic epithelial tumors, we performed high‐resolution spatial transcriptomic profiling (8‐µm pixel size) on tumor sections. Spatial transcriptomic data were normalized using the SCT framework prior to downstream analyses. Using RCTD to deconvolve spatial spots with our single‐cell reference, we projected malignant epithelial subpopulations and microenvironmental cell types onto the tissue (Figure 3A). Consistent with our single‐cell findings, CDH3 displayed a focal and patchy spatial expression pattern that was highly concordant with the inferred distribution of the C2_CDH3 malignant state, highlighting CDH3 as a defining feature of this spatially organized malignant epithelial population (Figure 3B,C).

**FIGURE 3:**
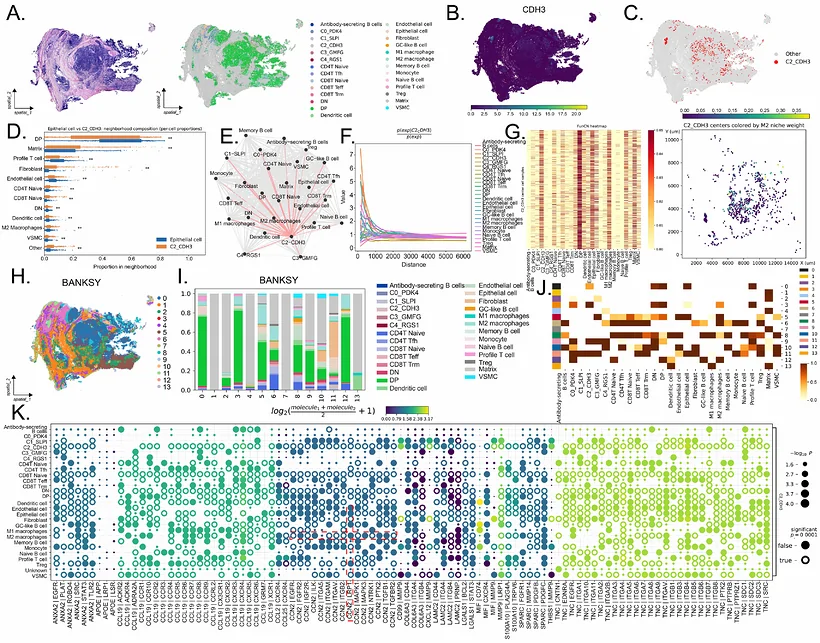
Spatial transcriptomics reveals a CDH3^+^ malignant niche in TETs. (A) Spatial projection of single‐cell‐defined malignant epithelial subpopulations and microenvironmental cell types onto tumor sections. (B) Spatial feature plot showing CDH3 expression across the tumor section. (C) Spatial distribution of the inferred CDH3^+^ malignant population. (D) Neighborhood composition analysis within a 120‐µm radius comparing regions enriched for CDH3^+^ malignant cells with neighborhoods surrounding non‐malignant epithelial cells. (E) Spatial interaction network analysis. (F) Distance‐dependent spatial co‐occurrence analysis. (G) FunCN analysis of the local cellular context surrounding CDH3^+^ malignant cells. (H) BANKSY spatially informed custering identifying 13 spatial ecological niches within the tumor. (I) Cell‐type composition of BANKSY‐defined niches. (J) Heatmap summarizing the enrichment of cell populations across BANKSY‐defined niches. (K) Dot plot of outgoing ligand–receptor interactions from CDH3^+^ malignant cells to surrounding cell populations. ^**^ *p* < 0.01 vs. the Epithelial cell.

We next quantified the local cellular context of malignant epithelium. Neighborhood analysis within a 120‐µm radius demonstrated that regions enriched for C2_CDH3 contained significantly higher proportions of M2 macrophages, fibroblasts and other stromal/immune components compared with neighborhoods surrounding non‐malignant epithelial cells (Figure 3D). Spatial interaction analysis revealed enhanced communication between C2_CDH3 malignant cells and myeloid compartments (Figure 3E), accompanied by preferential spatial co‐occurrence and predominantly short‐range interaction patterns (Figure 3F). FunCN analysis revealed marked spatial heterogeneity in the local context of C2_CDH3, with C2_CDH3‐centered regions exhibiting elevated M2 macrophage niche weights (Figure 3G), consistent with an immunosuppressive macrophage‐enriched microenvironment.

Application of the spatially informed clustering framework BANKSY identified 13 spatial ecological niches within tumors. Several niches showed co‐enrichment of C2_CDH3 malignant cells, M2 macrophages, and fibroblasts, together with additional stromal elements (Figure 3H–J), defining a recurrent CDH3^+^–M2 macrophage–fibroblast spatial ecosystem. Consistent with this spatial organization, analysis of outgoing ligand–receptor signals from C2_CDH3 malignant cells identified CCN2–ITGAV as a prominent epithelial–macrophage interaction, with stronger signaling toward M2 macrophages (Figure 3K), further supporting the presence of an immunosuppressive microenvironment associated with the C2_CDH3 malignant state.

To further validate these spatial relationships, we analyzed an independent public spatial transcriptomic dataset and mapped our single‐cell‐defined populations using cell2location. In this external dataset, the inferred C2_CDH3 distribution showed spatial concordance with M2 macrophages and fibroblasts, whereas M1 macrophages displayed a distinct pattern (Figure S3A–D). Moreover, spatial CDH3 expression closely recapitulated the mapped distribution of the C2_CDH3 population (Figure S3E). MISTy analysis further suggested that C2_CDH3‐enriched regions preferentially emitted immunoregulatory signals, including TGF‐β‐related pathways, both across individual sections and in the integrated analysis (Figure S3F,G), providing independent support for a spatially organized immunosuppressive niche.

### 2.5 Multiplex Immunofluorescence Validates a CDH3^+^ Tumor–M2 Macrophage Niche in TETs

To validate the tissue‐level organization of the CDH3‐associated niche, we performed multiplex immunofluorescence on a thymic epithelial tumor tissue microarray. Of the 95 patients included, 91 samples were successfully analyzed by HALO after quality control (Figure 4A). These samples included type A, AB, B1, B2, and B3 thymomas, thymic carcinomas, other rare tumor types, and normal thymus tissue (Figure 4B). Across these samples, the proportion of EPCAM^+^ epithelial cells varied by subtype (Figure 4C), whereas EPCAM^+^CDH3^+^ cells were largely absent in normal thymus and mainly detected in tumor tissues (Figure 4D). CD163^+^CD68^+^ M2 macrophages were also variably represented across pathological groups (Figure 4E). Representative images from different tumor subtypes are shown in Figure 4F.

**FIGURE 4:**
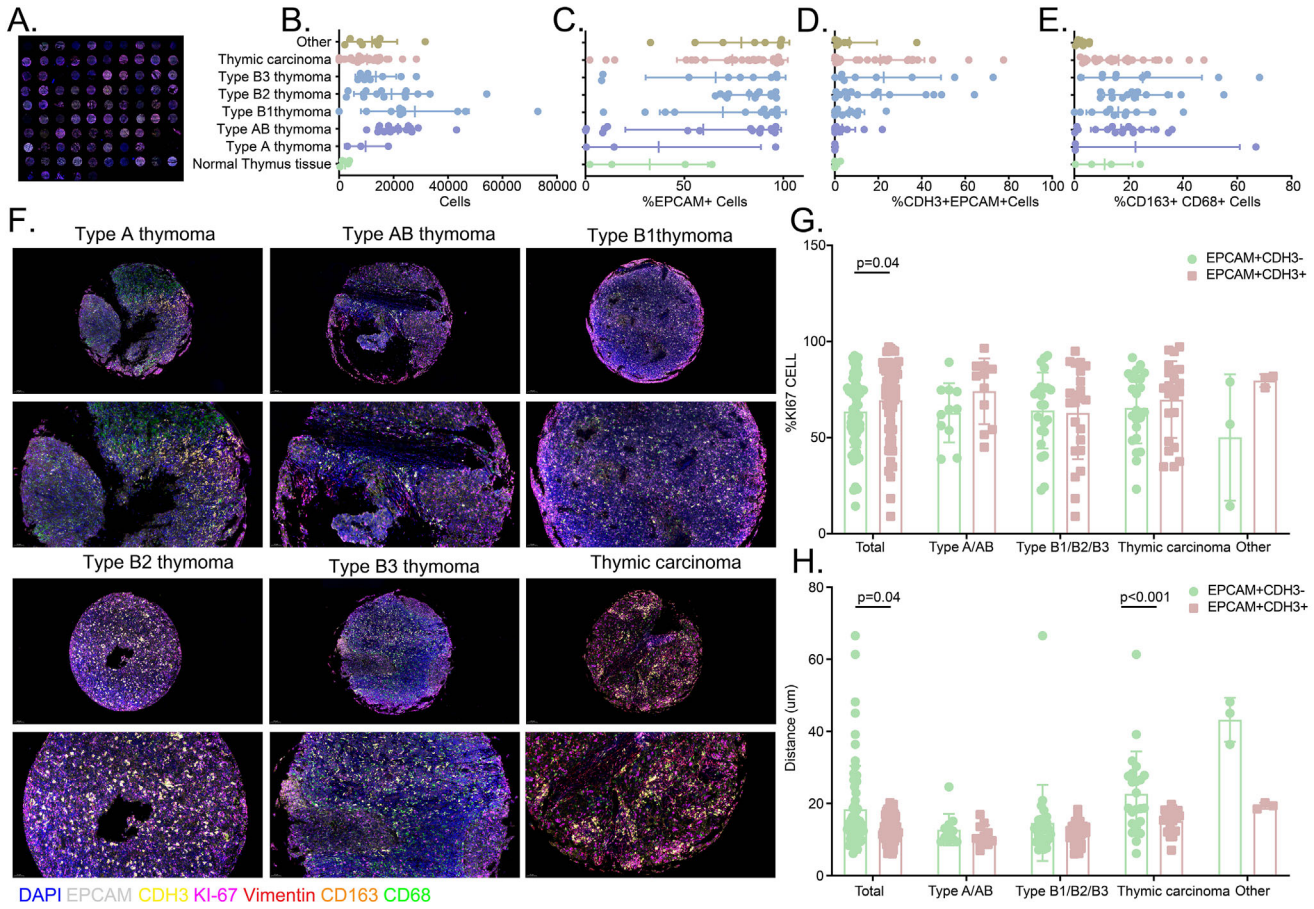
Multiplex immunofluorescence validates a CDH3^+^ tumor cell–M2 macrophage niche in TETs. (A) Tissue microarray overview for multiplex immunofluorescence analysis (n = 91 analyzable samples). (B) Total cell counts in analyzable samples grouped by pathological subtype. (C) Proportion of EPCAM^+^ epithelial cells across groups. (D) Proportion of EPCAM^+^CDH3^+^ epithelial tumor cells across groups. (E) Proportion of CD163^+^CD68^+^ M2 macrophages across groups. (F) Representative multiplex immunofluorescence images of major TET subtypes. DAPI, blue; EPCAM, gray; CDH3, yellow; Ki67, magenta; Vimentin, red; CD163, orange; CD68, green. (G) Comparison of the Ki67‐positive fraction between EPCAM^+^CDH3^+^ and EPCAM^+^CDH3^−^ epithelial cells. (H) Comparison of the mean distance from EPCAM^+^CDH3^+^ or EPCAM^+^CDH3^−^ epithelial cells to CD163^+^CD68^+^ M2 macrophages.

Quantitative analysis of quality‐controlled tumor samples showed that EPCAM^+^CDH3^+^ cells had a higher Ki67‐positive fraction than EPCAM^+^CDH3^−^ epithelial cells overall (Figure 4G), consistent with a more proliferative phenotype. In addition, EPCAM^+^CDH3^+^ cells exhibited a shorter mean distance to CD163^+^CD68^+^ M2 macrophages than EPCAM^+^CDH3^−^ epithelial cells (Figure 4H), indicating that CDH3‐positive tumor cells are spatially closer to M2 macrophages. These results provide tissue‐level support for a CDH3^+^ tumor cell–M2 macrophage immunosuppressive microenvironmental niche in TETs.

### 2.6 CDH3‐Associated Transcriptional Program Defines Molecular Subtypes With Distinct Genomic and Methylation‐associated Regulatory Features in TETs

To translate the CDH3^+^ malignant epithelial program into a bulk transcriptomic signature, we first performed hdWGCNA on the scRNA‐seq dataset to identify malignant cell‐associated co‐expression modules. Soft‐threshold selection supported robust scale‐free network construction (Figure S4A), and hierarchical clustering identified multiple gene modules across malignant epithelial cells (Figure S4B,C). Among these, the M7 module showed preferential expression in the C2_CDH3 subpopulation and contained a densely connected gene network centered on malignant epithelial features (Figure S4D).

We next intersected genes upregulated in C2_CDH3 with genes assigned to the M7 module, yielding a 68‐gene signature that was used to represent the CDH3‐associated malignant program (Figure S4E). Functional enrichment analysis of this gene set revealed prominent associations with oxidative phosphorylation, respiratory electron transport, and related metabolic pathways, together with Hallmark terms linked to apoptosis, estrogen response, IL6/JAK/STAT3 signaling, and inflammatory response (Figure S4F), suggesting that the CDH3^+^ malignant program is coupled to metabolic reprogramming and stress‐related signaling.

We then applied NMF to TCGA‐THYM bulk transcriptomes (n = 117) using this 68‐gene set. Rank survey and consensus metrics indicated that k = 2 provided the most stable and parsimonious solution (Figure 5A,B), resulting in two molecular clusters. The expression heatmap of the 68 genes revealed a clear dichotomy between the two groups (Figure 5C), and because CDH3 represented a prominent discriminator, we designated them as CDH3‐high and CDH3‐low groups (Figure 5D). Baseline clinicopathological characteristics of the two groups are summarized in Table S3. Kaplan–Meier analysis showed significantly worse overall survival in the CDH3‐high group than in the CDH3‐low group (Figure 5E). In multivariable Cox regression analysis adjusted for age, sex, race/ethnicity, histological subtype, and clinical stage, the CDH3‐high group remained independently associated with worse overall survival (HR = 26.28, 95% CI: 5.41–127.69; *p* < 0.0001; Table S4). Histologic classification also differed markedly between the two groups: B1/B2 thymomas predominated in the CDH3‐low group, whereas the CDH3‐high group was enriched for type A, B3, and C tumors (Figure 5F). InstaPrism‐based deconvolution also showed that the CDH3‐high group contained a larger proportion of C2_CDH3 cells (Figure 5G).

**FIGURE 5:**
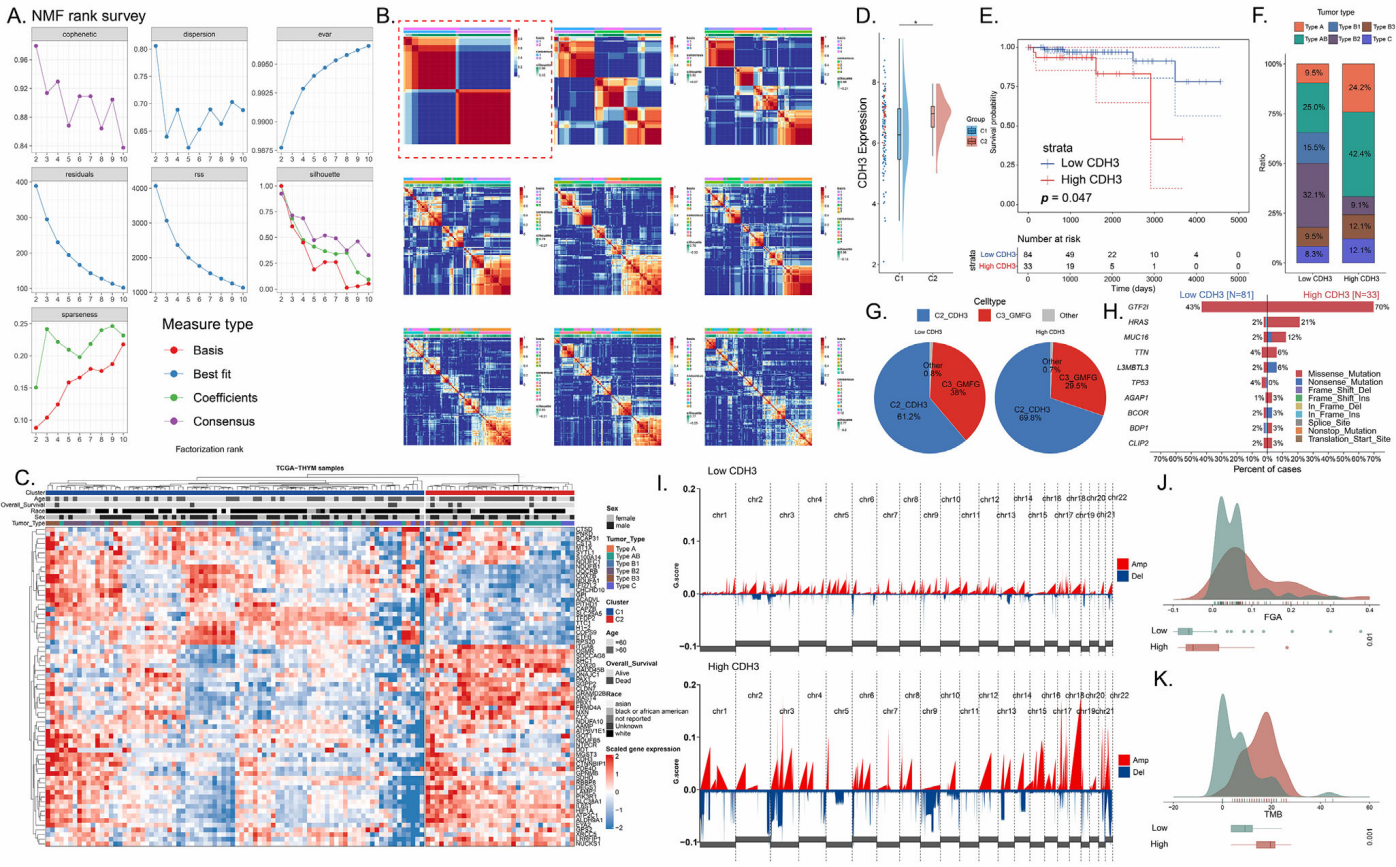
A CDH3^+^ malignant cell‐derived transcriptional program defines molecular subtypes in the TET cohort. (A) NMF rank survey for TCGA‐THYM bulk transcriptomes (n = 117) based on the 68‐gene CDH3^+^ malignant cells associated signature. (B) Consensus matrices for NMF clustering across different factorization ranks. (C) Heatmap of the 68‐gene signature across TCGA‐THYM samples. (D) Comparison of CDH3 expression between the two NMF‐defined clusters. (E) Kaplan–Meier overall survival analysis of the NMF‐defined subgroups. (F) Distribution of histologic subtypes of two groups. (G) InstaPrism‐based deconvolution of malignant epithelial states in the two bulk‐defined groups. (H) Comparison of recurrent somatic mutation frequencies. (I) Genome‐wide copy‐number alteration profiles of two groups. (J) Comparison of fraction of genome altered (FGA) between the two molecular subtypes. (K) Comparison of tumor mutational burden (TMB) between the two molecular subtypes. ^*^ *p* < 0.05 vs. C1.

To further validate the relationship between the CDH3‐associated program and histological subtype in an independent cohort, we performed bulk transcriptomic profiling and multiplex immunofluorescence analysis in 32 in‐house TET samples with detailed clinicopathological annotation, including age, sex, WHO subtype, invasion status, and necrosis status (Figure S5A). Multiplex immunofluorescence staining for DAPI, EPCAM, CDH3, and CD45, followed by HALO‐based quantitative image analysis, confirmed marked intertumoral heterogeneity in epithelial content and CDH3 positivity across WHO histological subtypes (Figure S5B–F). We next calculated ssGSVA scores using the subset of genes within the 68‐gene CDH3‐associated signature that were highly expressed in the CDH3‐high group. ROC analysis in TCGA‐THYM yielded an AUC of 0.855 and identified 0.46 as the optimal cutoff, which was then used to stratify the in‐house cohort into CDH3‐low‐like and CDH3‐high‐like groups (Figure S5G,H). Compared with the CDH3‐low‐like group, the CDH3‐high‐like group showed higher CDH3 expression, higher rates of tumor necrosis and invasion into surrounding tissues, and a distinct WHO subtype composition, including higher proportions of type A tumors and thymic carcinoma, lower proportions of type AB, B1, and B3 tumors, and a comparable proportion of type B2 tumors (Figure S5I–L). After normalization to EPCAM^+^ epithelial cells, CDH3‐high‐like tumors showed a higher overall EPCAM^+^CDH3^+^/EPCAM^+^ fraction than CDH3‐low‐like tumors. At the subtype level, numerically higher fractions were observed in type AB and type C tumors, whereas type B1, B2, and B3 tumors showed comparable or lower fractions in the CDH3‐high‐like group (Figure S5M). Consistently, deconvolution analysis in both TCGA‐THYM and the in‐house cohort showed higher estimated C2_CDH3 malignant epithelial‐state fractions in the CDH3‐high‐like groups across all analyzed WHO histological subtypes (Figure S5N).

Genomic profiling further distinguished these subtypes. The CDH3‐high group displayed higher mutation frequencies in genes including GTF2I, HRAS, and MUC16 (Figure 5H), consistent with subtype‐associated genomic differences in TCGA‐THYM. Copy‐number analyses showed that CDH3‐high tumors had more prominent amplification events and increased chromosomal instability, as reflected by elevated FGA/CIN scores (Figure 5I,J). In addition, tumor mutational burden (TMB) was higher in the CDH3‐high group (Figure 5K), indicating that this subgroup represents a more genomically unstable and mutation‐enriched TET subtype.

This pattern remained evident in the histotype‐stratified analysis, which revealed distinct copy‐number alteration profiles between CDH3‐high and CDH3‐low tumors across the available pathological categories (Figure S6A). At the single‐cell level, inferCNV analysis of malignant epithelial cells further identified C2_CDH3 as the subcluster with the highest inferred CNV score and a distinct large‐scale copy‐number alteration profile (Figure S6B,C). CDH3‐high tumors also exhibited a higher per‐sample SNV burden, altered proportions of the six major single‐base substitution classes, and distinct trinucleotide‐context spectra and COSMIC SBS signature exposures, with representative differences including increased contributions of SBS16 and SBS30 in the CDH3‐high group across the overall cohort and the analyzed histological categories (Figure S6D‐F). Histotype‐stratified analyses further showed similar differences in these mutational patterns across the available pathological categories (Figure S6G). TCGA‐THYM 450K methylation profiling showed significantly lower global DNA methylation levels in the CDH3‐high group, with variable effect sizes across histological categories (Figure S6H). Differentially methylated probes separated the two molecular groups and were associated with malignancy‐related pathways (Figure S6I,J). Binarized SCENIC analysis further revealed subcluster‐specific regulon activation patterns across malignant epithelial states, including a characteristic regulatory profile in C2_CDH3 cells (Figure S6K).

### 2.7 CDH3‐Associated TET Subgroups Exhibit Distinct Immune Microenvironment Features

To characterize immunoregulatory differences between the CDH3‐high and CDH3‐low groups, we profiled the tumor microenvironment using the IOBR R package. CDH3‐low tumors displayed higher enrichment of lymphocyte‐associated signatures, exemplified by elevated CD4_c3_Tfh and Th1_cells_Bindea_et_al scores, together with increased T‐cell–related signals such as CD8_c7_p_Tex and CD8_c13_Tn_TCF7, and higher B_cell_TIMER (Figure S7A). In contrast, CDH3‐high tumors exhibited a myeloid/stromal‐skewed microenvironment, characterized by increased Macrophages_M2_quantiseq and TAM_Peng_et_al signatures, alongside elevated fibroblast/CAF‐related programs including CAF_Peng_et_al and Fibroblasts_MCPcounter (Figure S7A).

At the program level, heatmap‐based profiling further indicated that CDH3‐high tumors were associated with coordinated upregulation of CAF‐related, TGF‐β–related, and EMT‐associated programs (Figure S7B). Consistently, quantitative comparisons confirmed significantly higher M2 macrophage scores (Macrophages_M2_quantiseq; Figure S7C), CAF scores (CAF_Peng_et_al; Figure S7D), and EMT scores (Figure S7E) in the CDH3‐high group. Histopathological observations were concordant with these transcriptome‐based patterns: representative sections from the CDH3‐low group showed more prominent immune infiltration, whereas CDH3‐high tumors tended to exhibit compact tumor nests with relatively sparse immune cell presence and a more stromal‐dominant appearance (Figure S7F,G).

### 2.8 CDH3 Promotes Malignant Phenotypes and Macrophage Crosstalk in Thymic Carcinoma

We first established a thymic carcinoma organoid model and observed increased CDH3 expression in the organoids (Figure S8A). Marker staining further confirmed the successful generation of thymic carcinoma organoids (Figure S8B), suggesting that CDH3 may be associated with the malignant phenotype of thymic epithelial tumors. To further assess the functional role and potential therapeutic relevance of this CDH3‐associated malignant program, we performed a series of loss‐of‐function assays in ThyL6 cells (Figure 6A). Efficient siRNA transfection and CDH3 knockdown were confirmed by fluorescence imaging and qPCR, respectively (Figure 6B). CCK‐8 assays showed that CDH3 silencing consistently suppressed ThyL6 cell proliferation from 24 to 96 h, as demonstrated by both growth curves and fold‐change analysis (Figure 6C,D). In addition, wound‐healing and Transwell migration assays revealed that CDH3 depletion significantly impaired the migratory capacity of ThyL6 cells (Figure 6E,F). Moreover, Matrigel‐coated Transwell invasion assays showed that CDH3 knockdown also reduced the invasive ability of ThyL6 cells (Figure 6G). Together, these findings indicate that CDH3 promotes the proliferative, migratory, and invasive phenotypes of thymic carcinoma cells.

**FIGURE 6:**
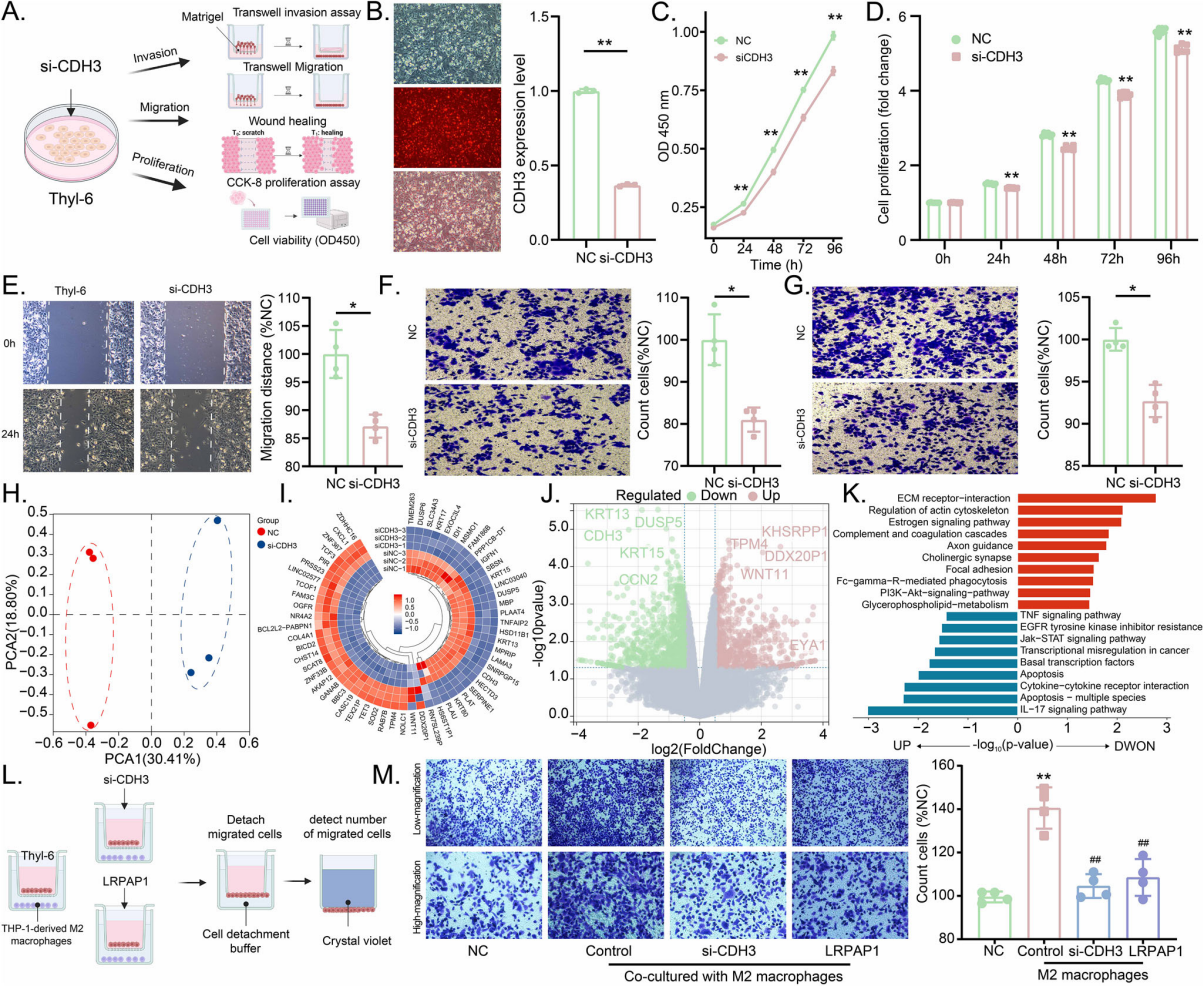
CDH3 promotes malignant phenotypes and macrophage crosstalk in thymic carcinoma. (A) Schematic overview of the loss‐of‐function assays performed in ThyL6 thymic carcinoma cells following CDH3 silencing. (B) Validation of siRNA transfection and CDH3 knockdown by fluorescence imaging and qPCR. (C–D) CCK‐8 assays showing reduced proliferation after CDH3 silencing. (E–F) Wound‐healing and Transwell migration assays showing impaired migration after CDH3 knockdown. (G) Matrigel‐coated Transwell assay showing reduced invasion after CDH3 knockdown. (H) Principal component analysis (PCA) of transcriptomic profiles from control and si‐CDH3 ThyL6 cells. (I) Heatmap showing the top 30 differentially expressed genes. (J) Volcano plot of differentially expressed genes following CDH3 silencing. (K) Pathway enrichment analysis of transcriptional changes induced by CDH3 silencing. (L) Schematic overview of the co‐culture assay used to assess macrophage‐associated effects on ThyL6 cell migration. (M) Representative images and quantification of Transwell migration in the co‐culture system. All quantitative cell‐function assays were performed with at least three biological replicates.^**^ *p* < 0.01 and ^*^ *p* < 0.05 vs. the NC group; ##p < 0.01 vs. the control group.

To further define the molecular consequences of CDH3 loss, we performed transcriptomic profiling of si‐CDH3 and control cells. PCA showed clear separation between the two groups, supporting substantial global transcriptional reprogramming upon CDH3 silencing (Figure 6H). Consistently, the top differentially expressed genes exhibited distinct expression patterns between control and si‐CDH3 cells (Figure 6I), and volcano plot analysis further illustrated extensive bidirectional transcriptional changes (Figure 6J). Notably, CCN2 expression decreased following CDH3 knockdown, linking CDH3 to the regulation of a candidate ligand implicated in macrophage crosstalk. Pathway enrichment analysis showed that si‐CDH3 cells were enriched for apoptosis‐ and TNF‐related pathways, whereas control cells preferentially exhibited enrichment of EMT, PI3K–Akt, and other protumorigenic signaling programs (Figure 6K), suggesting that CDH3 sustains an aggressive transcriptional state in thymic carcinoma cells.

We next examined whether CDH3 contributes to the prometastatic interaction between thymic carcinoma cells and macrophages. Co‐culture assays showed that THP‐1–derived M2 macrophages markedly enhanced ThyL6 cell migration. Importantly, this M2‐induced migratory effect was significantly attenuated either by CDH3 knockdown in ThyL6 cells or by LRP1 inhibition in the macrophage‐associated setting (Figure 6L,M). Together, these findings indicate that CDH3 promotes thymic carcinoma cell proliferation and migration and may facilitate tumor–macrophage crosstalk through the CCN2–LRP1 axis.

To further validate the CDH3‐specific tumor‐promoting effect in vivo, we established a xenograft model using ThyL6 cells stably transduced with control or CDH3‐targeting lentiviral shRNA (Figure S9A). Efficient transduction and CDH3 knockdown were confirmed by fluorescence imaging and qRT‐PCR (Figure S9B). Compared with the Sham group, CDH3 knockdown markedly suppressed xenograft tumor growth, as reflected by reduced tumor volume, tumor weight, and tumor index, without obvious effects on mouse body weight (Figure S9C–G). Histological and immunohistochemical analyses further confirmed tumor formation and reduced CDH3 expression in sh‐CDH3 xenografts (Figure S9H). These results provide CDH3‐specific in vivo evidence supporting the functional role of CDH3 in promoting thymic carcinoma growth.

### 2.9 Therapeutic Vulnerabilities Associated With the CDH3‐High Malignant Program in TETs

To assess the potential therapeutic implications of the CDH3‐associated malignant program, we first evaluated immunotherapy response using the TCGA cohort. TIDE analysis showed that the CDH3‐high risk group exhibited lower TIDE scores than the CDH3‐low group (Figure S10A), suggesting a higher predicted likelihood of benefiting from immune checkpoint blockade. Histotype‐stratified analyses showed a similar trend across the available pathological categories (Figure S10A). Given the clinical relevance of immune checkpoint blockade in thymic carcinoma, we further examined the independent pembrolizumab‐treated advanced thymic carcinoma cohort GSE181815. This cohort included 10 patients with thymic carcinoma, comprising five responders and five non‐responders. InstaPrism‐based deconvolution showed that the C2_CDH3 malignant epithelial state was more abundant in non‐responders than in responders (Figure S10B). These findings suggest that, although the CDH3‐high TCGA subgroup exhibited lower bulk TIDE scores, enrichment of the C2_CDH3 malignant state may be associated with an unfavorable response pattern in patients with advanced thymic carcinoma receiving pembrolizumab.

We next sought to identify candidate therapeutic compounds associated with this malignant program. Using Beyondcell across the epithelial compartment, we screened drugs associated with copyKAT‐defined tumor cells and prioritized compounds showing tumor‐related sensitivity patterns (Figure S10C). In parallel, drug sensitivity analysis in the TCGA cohort using oncoPredict identified multiple agents whose predicted IC50 values were negatively correlated with CDH3 expression (Figure S10D), suggesting increased sensitivity in tumors with higher CDH3 levels. Intersecting the candidates derived from these two complementary analyses yielded only two shared compounds, BMS‐754807 and MK‐2206 (Figure S10E), nominating them as the most robust candidates targeting the CDH3‐associated malignant phenotype.

To further evaluate the potential interaction between these compounds and CDH3, we performed molecular docking analysis. Both BMS‐754807 and MK‐2206 showed favorable binding to CDH3, with docking affinities of −7.7 kcal/mol and −8.6 kcal/mol, respectively (Figure S10F), supporting plausible direct interactions. Given its stronger predicted binding, MK‐2206 was further subjected to molecular dynamics simulation. Covariance and Gibbs free energy landscape analyses suggested a relatively stable conformational state of the MK‐2206–CDH3 complex during simulation (Figure S10G). Consistently, the RMSD, radius of gyration, hydrogen bond, and solvent accessible surface area profiles collectively supported the dynamic stability of the bound complex (Figure S10H–K). Together, these results nominate BMS‐754807 and MK‐2206 as candidate agents for targeting the CDH3‐associated malignant program in TETs.

### 2.10 In Vivo Validation of Candidate Therapies Targeting the CDH3‐Associated Malignant Program

To evaluate the therapeutic relevance of targeting CDH3‐associated signaling, we performed in vivo xenograft experiments using a thymic carcinoma model (Figure 7A). Macroscopic examination of excised tumors showed visibly reduced tumor burden in both treatment groups compared with controls (Figure 7B and Figure S11A). Consistently, longitudinal measurements demonstrated that treatment with either BMS‐754807 or MK‐2206 significantly suppressed tumor growth, as reflected by reduced tumor volumes over time (Figure 7C). In contrast, body weight remained largely unchanged across groups (Figure 7D), indicating limited systemic toxicity. At endpoint, both treatments resulted in a marked reduction in tumor weight (Figure 7E) and tumor index (tumor weight/body weight) (Figure 7F), further confirming their inhibitory effects on tumor progression.

**FIGURE 7:**
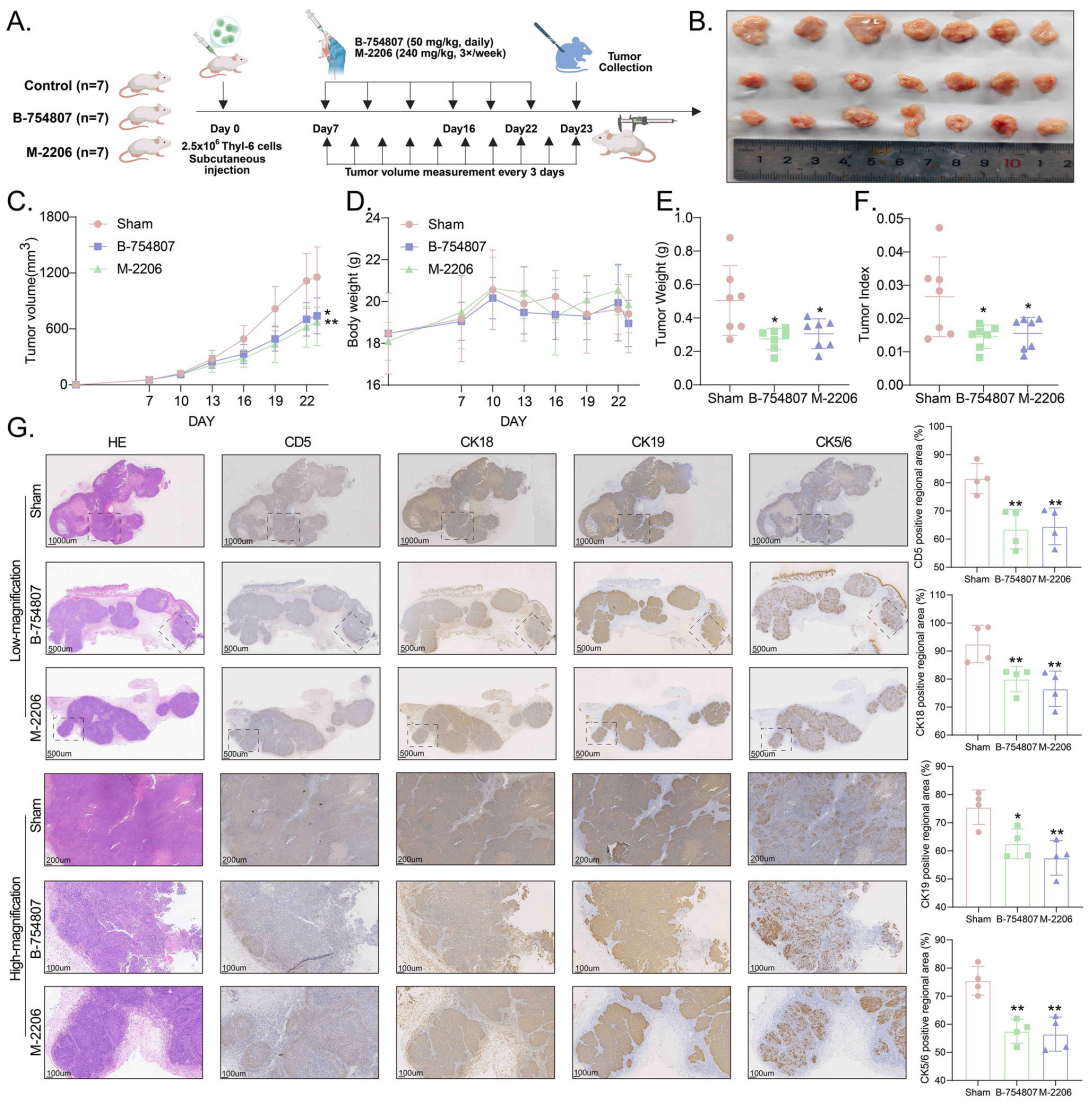
In vivo validation of candidate therapies targeting the CDH3‐associated malignant program in thymic carcinoma. (A) Schematic overview of the xenograft experiment (n = 7 mice per group). (B) Representative gross images of excised tumors from the Sham, BMS‐754807, and MK‐2206 groups at endpoint. (C) Tumor growth curves showing longitudinal tumor volume changes during treatment. (D) Body weight changes during treatment. (E) Comparison of tumor weight at endpoint among the three groups. (F) Comparison of tumor index (tumor weight/body weight) at endpoint among the three groups. (G) Representative H&E and immunohistochemical staining images of xenograft tumors from the Sham, BMS‐754807, and MK‐2206 groups at low and high magnification. ^**^ *p* < 0.01 and ^*^ *p* < 0.05 vs. the Sham group.

Histopathological analyses revealed that drug‐treated tumors exhibited decreased malignant features. Immunohistochemical staining demonstrated a consistent reduction in thymic epithelial tumor markers, including CD5, CK18, CK19, and CK5/6, in both treatment groups compared with controls (Figure 7G), indicating suppression of the epithelial malignant phenotype. To further explore treatment‐associated transcriptional changes, we performed RNA sequencing on three xenograft samples from each group. PCA showed clear separation among the sham, BMS‐754807, and MK‐2206 groups, indicating distinct drug‐induced transcriptional states (Figure S11B). Differential expression and pathway enrichment analyses further revealed treatment‐specific molecular responses. Compared with sham, BMS‐754807 treatment was associated with enrichment of pathways related to FASL/CD95L signaling, TNFSF receptor binding, and complement activation, whereas sham tumors were more strongly enriched for pathways involving extracellular matrix remodeling, β‐catenin signaling, senescence‐associated secretory phenotype, and epigenetic regulation (Figure S11C). Similarly, MK‐2206 treatment was associated with enrichment of complement‐related and microautophagy‐associated pathways, while sham tumors again showed relative enrichment of extracellular matrix organization, IL‐10 signaling, and β‐catenin‐related programs (Figure S11D).

### 2.11 Deep Learning–Based Pathology Modeling Captures CDH3‐Associated Malignant Morphology and Prognostic Risk

Deep learning models have become indispensable tools in modern medical image analysis, enabling the extraction of molecularly relevant signals from histopathological images that are not readily discernible by visual inspection alone. To determine whether the NMF‐defined CDH3‐related tumor subgroups could be recognized from routine histology, we established a weakly supervised computational pathology framework based on whole‐slide H&E images from the TCGA‐THYM cohort. WSIs were first segmented and tiled into image patches, from which patch‐level features were extracted using a pretrained ResNet‐50 encoder. These features were then aggregated at the slide level using an attention‐based multiple instance learning framework (CLAM_MB_MIL) to predict the NMF‐defined subgroup label (Figure 8A).

**FIGURE 8:**
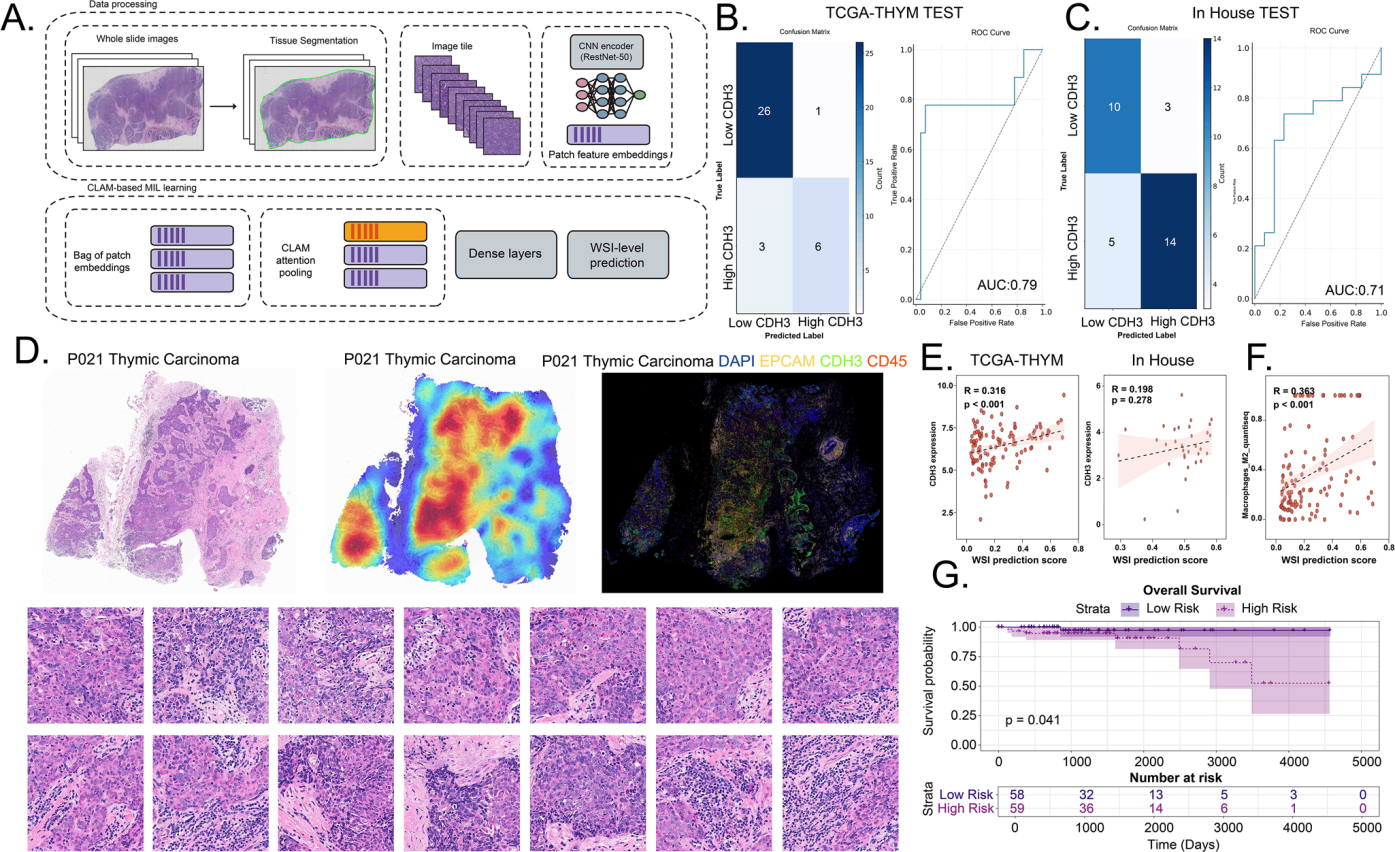
Deep learning–based pathology modeling captures CDH3‐associated malignant morphology and prognostic risk in TETs. (A) Schematic overview of the weakly supervised computational pathology framework. (B) Classification performance of the pathology model on the TCGA‐THYM held‐out test set (n = 36), shown by the confusion matrix and receiver operating characteristic (ROC) curve. (C) Classification performance of the pathology model on the independent in‐house validation cohort (n = 32), shown by the confusion matrix and ROC curve. (D) Spatial interpretability analysis in patient P021 from the in‐house cohort, showing the hematoxylin and eosin (H&E) image, the model‐derived attention heatmap, the corresponding multiplex immunofluorescence image stained for DAPI, EPCAM, CDH3, and CD45, and representative high‐attention histological patches identified by the model. (E) Correlation between the whole‐slide image (WSI) prediction score and CDH3 expression in TCGA‐THYM and the in‐house cohort. (F) Correlation between the WSI prediction score and M2 macrophage proportion across the TCGA‐THYM cohort. (G) Kaplan–Meier overall survival analysis stratified by pathology‐derived prediction score.

On the TCGA‐THYM held‐out test set, the model showed good discriminatory performance, with the confusion matrix indicating overall accurate classification of CDH3‐low and CDH3‐high tumors. ROC analysis yielded an AUC of 0.79, and the overall classification accuracy reached 0.89 (Figure 8B), indicating that H&E‐derived morphological features contain information capable of distinguishing CDH3‐related molecular subgroups defined from transcriptomic data. To further assess the generalizability of the model, we evaluated it in an independent in‐house cohort that was not used for model training or parameter tuning. In this external validation cohort, the model achieved an AUC of 0.71 and an overall accuracy of 0.75 (Figure 8C), supporting its ability to capture CDH3‐associated histological patterns beyond the TCGA‐THYM cohort.

To improve the histological interpretability of the model, we further examined a representative in‐house thymic carcinoma case, patient P021. The model‐derived attention heatmap highlighted spatially localized tumor regions on the H&E section. These high‐attention regions showed correspondence with the multiplex immunofluorescence image stained for DAPI, EPCAM, CDH3, and CD45, indicating that model‐highlighted areas were enriched for epithelial tumor regions with CDH3‐associated signals (Figure 8D). Representative high‐attention patches were selected from in‐focus tumor regions after excluding blurred areas, tissue folds, and likely artifacts, and showed interpretable tumor morphology. These findings support that the model captures spatially localized histomorphological features associated with the CDH3‐related malignant epithelial program. Besides, in representative TCGA cases from the high‐ and low‐CDH3 risk groups, slide‐level heatmaps likewise highlighted distinct high‐attention regions, and inspection of the corresponding image patches further revealed marked morphological differences between the two groups. Additional HoverNet‐based nuclear segmentation and virtual mIF reconstruction of these high‐attention regions suggested that high‐CDH3‐risk tumors exhibited closer spatial apposition between tumor cells and CD68‐positive macrophages, whereas this feature was less evident in low‐CDH3‐risk tumors (Figure S12A,B).

At the cohort level, the WSI prediction score was positively correlated with CDH3 expression in TCGA‐THYM (R = 0.316, *p* < 0.001; Figure 8E). In the independent in‐house cohort, the WSI prediction score showed a similar positive trend with CDH3 expression (Figure 8E). In TCGA‐THYM, the WSI prediction score was also positively correlated with the estimated M2 macrophage proportion (R = 0.363, *p* < 0.001; Figure 8F), further linking the pathology‐derived score to both the malignant epithelial program and the immunosuppressive microenvironment identified in our transcriptomic analyses. Finally, TCGA‐THYM patients were stratified into high‐ and low‐risk groups according to the pathology‐derived WSI prediction score. Baseline clinicopathological characteristics of the WSI‐defined risk groups are summarized in Table S3. Consistent with the biological aggressiveness associated with the CDH3‐related state, patients with higher WSI prediction scores exhibited significantly worse overall survival (Figure 8G). After adjustment for age, sex, race/ethnicity, histological subtype, and clinical stage, the high‐risk group remained associated with worse overall survival (HR = 9.22, 95% CI: 1.01–84.17; p = 0.049). When modeled as a continuous variable, each 0.1‐unit increase in the WSI prediction score was associated with an increased risk of all‐cause mortality (HR = 1.91, 95% CI: 1.27–2.88; *p* < 0.01; Table S4).

## 3 Discussion

In this study, we define a CDH3‐associated malignant epithelial program that integrates stemness, epithelial plasticity, and immunosuppressive niche formation in thymic epithelial tumors. Single‐cell and spatial analyses identified a CDH3^+^ malignant subpopulation at the root of tumor‐state trajectories and spatially linked it to M2 macrophages, with CCN2–LRP1 nominating a candidate epithelial–myeloid crosstalk axis. A CDH3‐associated transcriptional signature stratified TCGA‐THYM into subgroups with distinct survival, genomic instability, and immune contexture. Functional studies supported a role for CDH3 in tumor cell proliferation, migration, and macrophage‐associated signaling, whereas pharmacologic targeting suppressed tumor growth in xenograft models. Importantly, a deep learning pathology model captured CDH3‐associated morphology from routine H&E slides and predicted patient outcome. Collectively, these findings position CDH3 as a biomarker and functional mediator of aggressive TET biology and of associated M2 macrophage–rich immunosuppressive microenvironmental remodeling, while supporting pathology‐guided molecular stratification and therapeutic prioritization in TETs.

Although TETs have been increasingly characterized at the histologic, genomic, and immune levels, the malignant epithelial states that drive aggressive behavior and remodel the surrounding microenvironment remain poorly defined [15]. Our data identify CDH3 as a marker and functional mediator of a distinct malignant epithelial program in TETs. Single‐cell analyses positioned the CDH3‐associated subpopulation at the root of malignant‐state trajectories and linked it to poor outcome, genomic instability, and aggressive transcriptional features.The features of this CDH3‐associated state place it within the EMT continuum, which is now understood to span epithelial, mesenchymal, and hybrid epithelial/mesenchymal states in which tumor cells retain partial epithelial adhesion while progressively acquiring motility, stem‐like properties, and therapy resistance [16, 17]. Remodeling of adhesion molecules is a central component of this plasticity. As a classical type I cadherin, CDH3 regulates intercellular adhesion and epithelial plasticity and can modulate polarity and Wnt/EMT‐associated signaling through interactions with catenin family proteins [18, 19, 20]. Consistent with reports in other solid tumors linking CDH3 upregulation to invasion, metastasis, and therapeutic resistance [21, 22], our functional data showed that CDH3 silencing suppressed proliferation, migration, and protumorigenic transcriptional programs, including EMT‐ and PI3K‐Akt‐related signaling. Notably, the impact of this program extended beyond cell‐intrinsic behavior, as the CDH3‐associated malignant state was consistently embedded within a macrophage‐ and fibroblast‐rich spatial niche. Spatial transcriptomic, multiplex immunofluorescence, and cell‐cell communication analyses further converged on CCN2‐LRP1 as a candidate axis linking CDH3+ malignant epithelial cells to M2 macrophage‐associated signaling. Together, these findings indicate that the CDH3‐associated malignant program promotes TET progression by coupling aggressive epithelial behavior to local immune‐stromal remodeling.

TETs exhibit substantial biological heterogeneity, and histologic classification alone does not always distinguish indolent from aggressive disease [23]. Molecular subtyping provides a useful framework for refining prognostic assessment and linking tumor behavior to underlying genomic and microenvironmental features [24]. By translating the single‐cell‐defined CDH3‐associated malignant epithelial program into a 68‐gene bulk signature, we identified two TCGA‐THYM subgroups with distinct clinical and biological characteristics. The CDH3‐high subgroup showed worse survival, enrichment of more aggressive histologies, greater genomic instability, and a microenvironment marked by reduced lymphocyte‐associated signals together with increased M2 macrophage‐, CAF‐, TGF‐β‐, and EMT‐related programs. Together, these findings indicate that the CDH3‐associated malignant program promotes TET progression by coupling aggressive epithelial behavior to an M2 macrophage–associated immunosuppressive microenvironment, thereby providing additional biological and prognostic information within the established WHO histological framework.

Current systemic options for advanced TETs remain limited. For unresectable, recurrent, or progressive disease, chemotherapy remains the principal treatment, but responses are often transient, particularly in thymic carcinoma, and although immune checkpoint blockade has shown activity in a subset of patients, its use in TETs is constrained by a high risk of severe immune‐related toxicities [25, 26, 27]. Our data identify the CDH3‐associated malignant program as a therapeutically actionable vulnerability in this setting. CDH3 silencing suppressed thymic carcinoma cell proliferation, migration, and macrophage‐associated signaling, while integrative drug‐sensitivity analyses prioritized BMS‐754807 and MK‐2206 as candidate compounds linked to the CDH3‐high state. Both agents showed favorable in silico interaction with CDH3. Importantly, BMS‐754807 and MK‐2206 suppressed tumor growth in xenograft models. Together, these findings suggest that CDH3‐associated signaling may represent a therapeutically relevant target in TETs, with BMS‐754807 and MK‐2206 emerging as candidate agents for this aggressive subtype.

In current clinical practice, risk assessment for TETs still relies mainly on WHO histology and Masaoka‐Koga or TNM staging. Although these systems capture broad prognostic trends, they do not fully explain the marked variability in outcome and treatment response observed within the same clinicopathologic categories [28]. Large‐scale genomic and immune‐profiling studies have refined the biological landscape of TETs and revealed clinically relevant heterogeneity [29, 30], but their reliance on sequencing‐based or other high‐dimensional assays limits their routine use in pathology practice. Our findings suggest that part of this hidden biology is encoded in routine histomorphology. Using whole‐slide H&E images, we established a weakly supervised computational pathology model that accurately recognized the CDH3‐related molecular subgroup defined from bulk transcriptomic data. Importantly, the pathology‐derived prediction score was positively correlated with CDH3 expression and M2 macrophage abundance, and the model's spatial attention maps showed concordance with CDH3‐associated regions and tumor‐macrophage apposition. Patients with higher prediction scores also had significantly worse survival. Together, these results indicate that routine histology can capture the malignant and microenvironmental features of the CDH3‐associated program and support pathology‐guided molecular stratification as a clinically accessible approach for prognostic assessment in TETs.

Our study has several limitations. First, although we integrated multi‐omics data to elucidate the molecular heterogeneity of thymic epithelial tumors (TETs), the analyses relied heavily on publicly available datasets, which may introduce bias related to sample selection, data quality, and incomplete clinical annotation. Given the limited number of mortality events in TCGA‐THYM, the prognostic estimates should be interpreted cautiously. In addition, survival data from the in‐house cohort are still being collected and curated, and the number of thymoma cases within each WHO subtype remains limited. Therefore, the prognostic value of CDH3 status and subtype‐specific epithelial‐normalized CDH3 patterns require further validation in larger, subtype‐balanced cohorts with extended follow‐up. Matched single‐cell DNA and epigenomic data were also unavailable, and future studies are needed to directly validate the genomic and epigenetic differences between CDH3‐positive and CDH3‐negative epithelial cells. Second, while we performed multi‐layer computational validation and conducted functional experiments confirming the role of CDH3, in vitro assays were limited to specific TET cell models, and in vivo experiments were performed using a single xenograft model. Additional validation across multiple TET subtypes and independent cohorts is needed to strengthen the generalizability of our findings. Third, although our pathology‐anchored framework enables effective stratification based on routine histology, the model was primarily trained on TCGA‐THYM slides, and its performance may vary across different institutions due to variations in staining procedures, scanning platforms, and pathological workflows. External, multi‐center validation is therefore essential before clinical deployment. Furthermore, although we mapped CDH3‐associated vulnerabilities and identified candidate small‐molecule inhibitors, mechanistic studies on CDH3 signaling—especially its interactions within EMT/CSC programs and the tumor microenvironment—remain incomplete and require deeper exploration. Lastly, while we focused on CDH3 as a key driver, TET progression is governed by complex regulatory networks, and future work should integrate additional genomic, spatial, and immunological features to construct more comprehensive clinical decision‐support systems.

In conclusion, we define a CDH3‐associated malignant epithelial program in TETs that links epithelial plasticity, stemness, and the formation of an M2 macrophage–associated immunosuppressive niche. Multi‐omic and histopathologic analyses identify CDH3 as a biomarker and functional driver of aggressive disease, associated with poor prognosis, genomic instability, and a macrophage/fibroblast‐rich tumor microenvironment. Functional and pharmacologic studies further support CDH3 as a potentially targetable mediator of aggressive TET biology, with pharmacologic inhibition showing antitumor effects in xenograft models.

## 4 Experimental Section

### 4.1 Data Sources

Publicly available datasets were obtained from TCGA and GEO/GSA. Histopathology whole‐slide images, bulk RNA‐seq data, and matched clinical information for thymic epithelial tumors (TETs) were downloaded from TCGA‐THYM through the Genomic Data Commons portal (https://portal.gdc.cancer.gov/). After quality control, 117 cases with complete transcriptomic and clinical data were retained, corresponding to 177 whole‐slide images. Single‐cell RNA‐seq data for TETs were obtained from the Genome Sequence Archive (HRA002334). An independent pembrolizumab‐treated cohort with transcriptomic and response data (GSE181815) was also downloaded from GEO.

Clinical specimens were collected at Shanghai Pulmonary Hospital. An independent in‐house cohort of 32 TET patients was included for bulk transcriptomic profiling, multiplex immunofluorescence staining, and clinicopathological validation, including type A thymoma (*n* = 3), type AB thymoma (*n* = 4), type B1 thymoma (*n* = 4), type B2 thymoma (*n* = 5), type B3 thymoma (*n* = 4), and thymic carcinoma (*n* = 12). One TET sample (Type B2 Thymoma) was used for frozen‐section molecular and histopathological analyses, and one thymic carcinoma sample was used for organoid culture. Written informed consent was obtained from all patients. The study was approved by the Institutional Review Board of Shanghai Pulmonary Hospital (approval number: K26‐019) and conducted in accordance with the Declaration of Helsinki.

### 4.2 Single‐Cell RNA‐seq Data Processing and Downstream Analyses

Raw sequencing data were processed with CellRanger (10x Genomics) to generate gene expression matrices, and downstream analyses were performed in Seurat (v5.1) [31]. Cells expressing fewer than 500 genes, with fewer than 500 UMIs, or with >25% mitochondrial transcripts were excluded. After quality control, data were normalized and scaled using NormalizeData and ScaleData. Single‐cell datasets from TETs (HRA002334) were integrated with Harmony to correct batch effects [32]. Highly variable genes identified by FindVariableFeatures were used for principal component analysis, followed by k‐nearest neighbor graph construction with FindNeighbors, clustering with FindClusters (resolution = 0.8), and visualization by UMAP. Cell types were annotated according to canonical marker genes from previous studies.

The epithelial compartment was extracted and analyzed with CopyKAT to infer large‐scale copy number variation profiles, and aneuploid epithelial cells were defined as malignant [33]. Malignant cell populations associated with clinical phenotypes were identified using Scissor by integrating malignant single‐cell transcriptomes with bulk RNA‐seq and survival data from TCGA‐THYM [34]. Cell‐state dynamics were analyzed with Monocle2 (v2.26.0) using the DDRTree algorithm [35], together with CytoTRACE [36] and scTour [37]. Hallmark pathway activities were scored in individual cells using AddModuleScore, and subpopulation‐level scores were visualized after row‐wise Z‐score scaling. Transcriptional regulatory network analysis was performed using the pySCENIC workflow, including GRNBoost2, RcisTarget, and AUCell [38]. Simulated knockout analysis was conducted with scTenifoldKnk (v1.2.0), and perturbed genes were further analyzed for functional enrichment using Enrichr platform (https://maayanlab.cloud/Enrichr/).

### 4.3 Cell–Cell Communication Analysis

Cell–cell communication was analyzed using the CellChat R package (v1.1.1) [39]. Communication probabilities and signaling networks were computed with computeCommunProb, filterCommunication, and computeCommunProbPathway. Aggregated networks were visualized using netVisual_circle and netVisual_bubble to display interaction strength and key ligand–receptor pairs. To further infer ligand‐driven downstream effects in specific sender–receiver pairs, NicheNet analysis was performed. NicheNet prioritizes active ligands by integrating prior knowledge of ligand–receptor, intracellular signaling, and transcriptional regulatory networks, and links sender‐cell ligands to target genes in receiver cells [40].

### 4.4 Spatial Transcriptomics Analysis

Probe‐based spatial transcriptomic profiling was performed on FFPE TET sections using the DynaSpatial FFPE Spatial Gene Expression Kit (Dynamic Biosystems, Suzhou, China) according to the manufacturer's instructions [41]. FFPE sections (5 µm) were mounted on adhesive slides, deparaffinized, stained with hematoxylin and eosin, and imaged using a Pannoramic MIDI II digital slide scanner (3DHISTECH, Hungary). After decrosslinking, transcriptome‐targeting probes were hybridized and ligated, released from tissue sections, captured on spatially barcoded DynaSpatial slides using the Dynablot system, and subjected to probe extension. Sequencing libraries were prepared according to the manufacturer's protocol, quantified by Qubit 4.0, assessed using an Agilent 4150 TapeStation, and sequenced in paired‐end 150‐bp mode on the Illumina NovaSeq X Plus or MGI T7 platform.

Spatial count matrices were processed in R and normalized using SCTransform. Spatial deconvolution was performed with RCTD using the single‐cell RNA‐seq dataset generated in this study as the reference. Neighborhood composition was quantified within a 120‐µm radius around each spatial location. Spatial co‐occurrence, proximity, and interaction‐distance analyses were performed based on deconvolved cell‐state distributions. FunCN was used for local niche modeling [42], and BANKSY was applied for spatial domain identification. Ligand‐receptor analysis was performed using spatially assigned cell‐state abundances. An independent public spatial transcriptomic dataset [43] was analyzed separately using cell2location for reference‐based mapping and MISTy for spatial signaling modeling.

### 4.5 Multiplex Immunofluorescence Staining and Halo Image Analysis

Multiplex immunofluorescence (mIHC) was performed on a thymic epithelial tumor tissue microarray using a tyramide signal amplification (TSA)‐based protocol according to the manufacturer's instructions. Formalin‐fixed paraffin‐embedded sections were deparaffinized, rehydrated, subjected to heat‐induced antigen retrieval, and blocked for endogenous peroxidase activity and nonspecific binding. Primary antibodies were incubated at 4°C overnight, followed by sequential HRP‐conjugated secondary antibody and TSA fluorophore staining cycles with microwave stripping between cycles. Nuclei were counterstained with DAPI. Antibodies used included EPCAM, CDH3, CD163, CD68, Ki67, and Vimentin.

Images were analyzed in HALO (Indica Labs) using the HighPlex FL and Spatial Analysis modules. After exclusion of low‐quality areas, cells were segmented by DAPI and phenotyped by marker‐intensity thresholds. EPCAM+CDH3+ cells were annotated as CDH3+ tumor cells, EPCAM+CDH3‐ cells as other epithelial tumor cells, and CD163+CD68+ cells as M2 macrophages. Nearest‐neighbor, proximity, and cell‐density metrics were extracted for statistical analysis.

For the in‐house validation cohort, multiplex immunofluorescence staining was performed using DAPI, EPCAM, CDH3, and CD45. HALO‐based quantification was used to calculate total segmented cells, EPCAM^+^ epithelial‐cell fractions, CDH3^+^ cell fractions among all cells, and epithelial‐normalized CDH3 positivity, defined as EPCAM^+^CDH3^+^ cells divided by EPCAM^+^ epithelial cells.

### 4.6 High‐Dimensional Weighted Gene Co‐Expression Network Analysis (hdWGCNA)

hdWGCNA was conducted to identify co‐expression modules [44]. After metacell construction, a scale‐free network was built, and gene modules were defined through hierarchical clustering. Module eigengenes were computed to assess module–trait correlations, and hub genes were identified based on intramodular connectivity (kME).

### 4.7 Deconvolution Analysis

Bulk transcriptomic deconvolution was performed using InstaPrism [45]. Single‐cell RNA‐seq data were used to generate the reference expression matrix, and normalized bulk RNA‐seq data were deconvolved to estimate cell‐type proportions for each sample.

### 4.8 NMF‐Based Molecular Subtyping and Genomic Profiling

TCGA‐THYM bulk RNA‐seq data were analyzed using the NMF R package based on the expression matrix of the CDH3‐associated signature genes. The brunet algorithm was run for 100 iterations at each rank, and ranks 2–10 were evaluated. The optimal cluster number was determined according to cophenetic correlation, dispersion, and silhouette width. Overall survival was analyzed using the Kaplan‐Meier method, and histologic subtype annotations were obtained from the matched clinical data. Somatic mutation, copy‐number alteration, fraction of genome altered (FGA), chromosomal instability (CIN), and tumor mutational burden (TMB) data were retrieved from TCGA‐THYM and compared across the NMF‐defined subtypes.

For ssGSVA analysis, genes upregulated in the TCGA CDH3‐high group within the 68‐gene CDH3‐associated signature were used as the input gene set. Sample‐level ssGSVA enrichment scores were calculated from normalized bulk RNA‐seq expression matrices and used for downstream validation analyses.Copy‐number alteration profiles, FGA, CIN, and TMB were analyzed using TCGA‐THYM data. Single‐cell CNV patterns were inferred using inferCNV. SNV burden, six single‐base substitution classes, trinucleotide‐context spectra, COSMIC SBS signature exposures, TCGA 450K DNA methylation profiles, differentially methylated probes, and binarized SCENIC regulon activity were also analyzed.

### 4.9 Comprehensive Immunogenomic Profiling and Immune Response Prediction

Immune microenvironment profiling of the CDH3‐high and CDH3‐low subgroups was performed using the IOBR R package [46]. Bulk RNA‐seq expression matrices were analyzed in ssGSEA mode to quantify immune cell infiltration and microenvironment‐related programs, including lymphoid, myeloid, and CAF. Signature scores were extracted from the normalized expression matrix and compared between subgroups using two‐sided Wilcoxon rank‐sum tests. Immune landscape patterns were visualized by z‐score‐scaled heatmaps with hierarchical clustering. Representative histopathological sections were reviewed in parallel with the transcriptome‐based immune profiling.

### 4.10 Cell Functional Assays

The human thymic carcinoma cell line ThyL‐6 was originally established at the University of Fukui [47] and was formally obtained in this study through the International Patent Organism Depositary (IPOD, NITE, Japan) via the official distribution process coordinated by the University of Fukui. ThyL‐6 cells were cultured in RPMI‐1640 supplemented with 10% fetal bovine serum, 100 U/mL penicillin, and 100 µg/mL streptomycin at 37°C with 5% CO2. THP‐1 cells were maintained under standard culture conditions. CDH3 knockdown was performed using CDH3‐targeting siRNA. For stable knockdown, ThyL‐6 cells were transduced with a CDH3‐targeting lentiviral shRNA to generate sh‐CDH3 cells for subsequent in vivo xenograft experiments. Knockdown efficiency was assessed by fluorescence observation and qRT‐PCR (primer sequences and siRNA/shRNA target sequences are listed in Tables S1 and S2). Cell proliferation, migration, and invasion were evaluated by CCK‐8, wound‐healing/Transwell migration, and Matrigel‐coated Transwell invasion assays, respectively. THP‐1‐derived M2 macrophages were generated using PMA, IL‐4, and IL‐13 for co‐culture experiments.

### 4.11 Drug Sensitivity Analysis

Immunotherapy response in TCGA‐THYM was predicted using TIDE [48], and malignant epithelial states were mapped to the pembrolizumab‐treated cohort GSE181815 using InstaPrism. Drug sensitivity was estimated at the single‐cell level with BeyondCell (v1.0) [49] and at the bulk level in TCGA‐THYM with oncoPredict, and overlapping candidates were selected. Molecular docking was performed using AutoDockTools (v1.5.7) [50]. Molecular dynamics simulation of the MK‐2206‐CDH3 complex was then conducted, and trajectory stability was evaluated using RMSD, radius of gyration, hydrogen bond, solvent‐accessible surface area, covariance, and Gibbs free energy landscape analyses.

### 4.12 In Vivo Xenograft Experiments

Subcutaneous xenograft experiments were performed in immunodeficient mice using ThyL6 thymic carcinoma cells. Cells were injected subcutaneously into the flank, and mice were randomized into sham, BMS‐754807, and MK‐2206 groups when tumors reached a comparable size. The indicated compounds or vehicle were administered according to the treatment schedule. In parallel, CDH3‐specific knockdown xenograft experiments were performed using ThyL6 cells stably transduced with control or CDH3‐targeting lentiviral shRNA. Tumor length and width were measured with digital calipers at the indicated time points, and tumor volume was calculated as 0.5 × length × width^2^. Body weight was recorded throughout the experiment. At the endpoint, mice were euthanized and tumors were excised and weighed. Tumor index was calculated as tumor weight/body weight. All animal experiments were approved by the Ethics Committee of Shanghai Pulmonary Hospital (approval number: K26‐019) and conducted in accordance with institutional guidelines for animal care and use

### 4.13 Patient‐Derived Organoid Culture and Drug Treatment

Fresh thymic carcinoma tissue was minced, enzymatically dissociated, embedded in Matrigel, and cultured in organoid medium at 37°C with 5% CO2. Medium was changed every 2–3 days.

### 4.14 Immunohistochemistry

Immunohistochemistry (IHC) was performed on paraffin sections using standard procedures. After deparaffinization, rehydration, and antigen retrieval, endogenous peroxidase activity was blocked and sections were incubated with primary antibodies at 4°C overnight. After incubation with HRP‐conjugated secondary antibodies, signals were visualized with DAB and sections were counterstained with hematoxylin. Stained slides were scanned and evaluated microscopically. Primary antibodies included CD5, CK18, CK19, CK5/6, BCL‐2, GLUT1, PanCK, and P40, as indicated for each experiment.

### 4.15 RNA Sequencing and Transcriptomic Analysis

RNA sequencing was performed on CDH3‐silenced and control ThyL6 cells, as well as xenograft tumors from the sham, BMS‐754807, and MK‐2206 groups. Total RNA was extracted using TRIzol reagent (Invitrogen). RNA concentration and purity were measured with a NanoDrop 2000 spectrophotometer (Thermo Scientific), and RNA integrity was assessed using an Agilent 2100 Bioanalyzer (Agilent Technologies). Libraries were prepared with the VAHTS Universal V6 RNA‐seq Library Prep Kit according to the manufacturer's instructions and sequenced on the Illumina NovaSeq 6000 platform in paired‐end 150‐bp mode. Raw FASTQ files were processed with fastp to remove low‐quality reads, and clean reads were aligned to the reference genome using HISAT2. Gene‐level read counts were generated with HTSeq‐count. Principal component analysis was performed in R. Differential expression analysis was conducted using limma, with *p* < 0.05 and fold change >1.4 or <0.7 as the cutoff. Functional interpretation was performed by KEGG and Reactome enrichment analyses, together with gene set enrichment analysis.

### 4.16 Computational Pathology Modeling Based on Whole‐Slide Images

Whole‐slide images (WSIs) were processed using an OpenSlide‐based workflow. Tissue regions were segmented from the background, and each slide was tiled into non‐overlapping image patches. Patch‐level visual features were extracted using a pretrained ResNet‐50 convolutional neural network, and the resulting embeddings were used as input for downstream multiple instance learning. Slide‐level classification was performed using clustering‐constrained attention multiple instance learning (CLAM), with patch embeddings aggregated by the CLAM_MB_MIL architecture for binary classification. Each slide was assigned the subgroup label defined by transcriptome‐based NMF stratification, and the TCGA‐THYM dataset was randomly split into training, validation, and test sets at a ratio of 0.5:0.3:0.2. The trained model was further evaluated in the independent in‐house cohort using the same WSI processing, patch extraction, feature extraction, and slide‐level prediction pipeline, with Reinhard color normalization applied before feature extraction. Model performance was evaluated using ROC curves, AUC, accuracy, and confusion matrix analysis. High‐attention regions were further analyzed using HoVer‐Net for nuclei segmentation and classification [51]. Virtual multiplex immunofluorescence reconstruction was performed with GigaTIME, a multimodal model for translating H&E images into virtual mIF images, and the reconstructed marker channels were used for spatial visualization of microenvironment‐related features [52]. Finally, attention scores were projected back to tissue space to generate slide‐level heatmaps, and the maximum slide‐level prediction score was retained as the patient‐level score when multiple WSIs were available. Patient‐level scores were used for correlation and survival analyses [53].

### 4.17 Cox Regression Analysis

Cox proportional hazards regression analyses were performed to evaluate the prognostic associations of CDH3‐defined molecular subtype and pathology‐derived WSI prediction score with overall survival. Univariable and multivariable Cox models were constructed using the survival package in R. Multivariable models were adjusted for age, sex, race/ethnicity, histological subtype, and clinical stage. Hazard ratios (HRs) and 95% confidence intervals (CIs) were reported. For continuous‐variable analysis, the WSI prediction score was modeled per 0.1‐unit increase. A two‐sided *p* < 0.05 was considered statistically significant.

### 4.18 Statistical Analysis

All analyses were performed in R (v4.4.2). Group differences were evaluated using the Student's t‐test or Wilcoxon rank‐sum test, and correlations were assessed by Spearman analysis. Survival curves were compared using the Kaplan–Meier method with the log‐rank test, with two‐tailed *p* < 0.05 considered statistically significant.

## Author Contributions

**Guofang Zhao**: funding acquisition, methodology. **Long Xu**: formal analysis, supervision. **Jingyu Chen**: methodology. **Tao Wang**: formal analysis, methodology, validation. **Lang Xia**: writing – original draft, supervision, software, investigation, validation. **Juemin Yu**: supervision, formal analysis. **Yue Zhao**: supervision, resources, data curation, writing – review and editing, funding acquisition, conceptualization, project administration. **Yuntao Feng**: writing – original draft, investigation, data curation, methodology, visualization, software. **Lei Zhang**: writing – original draft, visualization, validation, investigation. **Yuzhou Wang**: methodology. **Yunlang She**: supervision, data curation, resources. **Junqi Wu**: supervision, writing – review and editing, resources, data curation. **Deping Zhao**: funding acquisition, writing – review and editing, supervision, project administration, resources. **Chang Chen**: project administration, resources, supervision, writing – review and editing, funding acquisition, conceptualization, methodology.

## Funding

This study was funded by the National Natural Science Foundation of China (No. 82573891), the Natural Science Foundation of Shanghai (25SF1901800), the Ningbo Top Medical and Health Research Program (No.2022030208) and the Huadong Medicine Joint Funds of the Zhejiang Provincial Natural Science Foundation of China (No. LHDMD23H160001).

## Ethics Statement

This study was conducted in accordance with the Declaration of Helsinki. Clinical specimens were collected from Shanghai Pulmonary Hospital, and written informed consent was obtained from all patients prior to sample collection. The study protocol was approved by the Institutional Review Board of Shanghai Pulmonary Hospital (approval number: K26‐019). All animal experiments were performed in accordance with institutional guidelines and were approved by the Ethics Committee of Shanghai Pulmonary Hospital (approval number: K26‐019). Five‐ to six‐week‐old male BALB/c nude mice used for xenograft experiments were obtained from the Animal Laboratory of Shanghai Pulmonary Hospital.

## Consent

All authors consent to the publication of the article.

## Conflicts of Interest

The authors declare no conflicts of interest.

## Supporting information




**Supporting File**: advs77153‐sup‐0001‐SuppMat.docx.

## Data Availability

Publicly available datasets used in this study include bulk RNA‐seq data, whole‐slide images, and matched clinical information for thymic epithelial tumors from TCGA‐THYM through the Genomic Data Commons (GDC) portal; single‐cell RNA‐seq data for thymic epithelial tumors from the Genome Sequence Archive under accession HRA002334; the independent pembrolizumab‐treated cohort from GEO under accession GSE181815; and the public spatial transcriptomic dataset used for external validation from Yasumizu et al. (raw data: JGAS000672; processed data: E‐GEAD‐747; figsharehttps://doi.org/10.6084/m9.figshare.25052546). Additional data generated or analyzed during this study are available from the corresponding authors upon reasonable request.
