# Supplementary material for: Multi‐Omics Integration Identifies a CDH3‐Associated Malignant Epithelial State and Immunosuppressive Niche to Predict Prognosis in Thymic Epithelial Tumors
Source: Adv Sci (Weinh). 2026 Aug 15:e77153. Online ahead of print. doi: 10.1002/advs.77153 (PMC13477261; doi:10.1002/advs.77153)
Supplement: Supplementary file 1 [file ADVS-9999-e77153-s001.docx]

**Supplementary Table:**

**Table S1**. **Primer sequences employed for qRT-PCR in this study**

| Gene | Forward primer sequence (5’ −> 3’) | Reverse primer sequence (5’ −> 3’) | Species |
| --- | --- | --- | --- |
| CDH3 | GAGATTGGGTGGTTGCTCCA | CAACAACCAGCCTGTCTCCT | Human |
| GAPDH | GTCTTCACCACCATGGAGAA | AGGGACTCGAAAGTCCCAAA | Human |

**Table S2**. **siRNA and lentiviral shRNA target sequences used for CDH3 knockdown in this study**

| Reagent | Target gene | Target sequence (5′ → 3′) | Species |
| --- | --- | --- | --- |
| si-CDH3 | CDH3 | GGCTGGTTGTTGTTGAATA | Human |
| sh-CDH3 | CDH3 | GGCTGGTTGTTGTTGAATA | Human |

**Table S3**. **Baseline clinicopathological characteristics of the TCGA-THYM cohort.**

|  | **Total** | **Low CDH3** | **High CDH3** | **P-value** | **Low Risk** | **High Risk** | **P-value** |
| --- | --- | --- | --- | --- | --- | --- | --- |
|  | **(N=117)** | **(N=84)** | **(N=33)** |  | **(N=58)** | **(N=59)** |  |
| Age, (years) , n (%) |  |  |  |  |  |  |  |
| <60 | 57 (48.7%) | 46 (54.8%) | 11 (33.3%) | 0.052 | 34 (58.6%) | 23 (39.0%) | 0.041 |
| >=60 | 59 (50.4%) | 37 (44.0%) | 22 (66.7%) |  | 23 (39.7%) | 36 (61.0%) |  |
| Missing | 1 (0.9%) | 1 (1.2%) | 0 (0%) |  | 1 (1.7%) | 0 (0%) |  |
| Sex (%) |  |  |  |  |  |  |  |
| Female | 57 (48.7%) | 39 (46.4%) | 18 (54.5%) | 0.559 | 30 (51.7%) | 27 (45.8%) | 0.645 |
| Male | 60 (51.3%) | 45 (53.6%) | 15 (45.5%) |  | 28 (48.3%) | 32 (54.2%) |  |
| Race/ethnicity, n (%) |  |  |  |  |  |  |  |
| White | 97 (82.9%) | 70 (83.3%) | 27 (81.8%) | 0.479 | 51 (87.9%) | 46 (78.0%) | 0.036 |
| Black or African American | 6 (5.1%) | 5 (6.0%) | 1 (3.0%) |  | 5 (8.6%) | 1 (1.7%) |  |
| Asian | 12 (10.3%) | 8 (9.5%) | 4 (12.1%) |  | 2 (3.4%) | 10 (16.9%) |  |
| not reported | 1 (0.9%) | 0 (0%) | 1 (3.0%) |  | 0 (0%) | 1 (1.7%) |  |
| Unknown | 1 (0.9%) | 1 (1.2%) | 0 (0%) |  | 0 (0%) | 1 (1.7%) |  |
| Pathological classification, n (%) |  |  |  |  |  |  |  |
| Type A | 16 (13.7%) | 8 (9.5%) | 8 (24.2%) |  | 2 (3.4%) | 14 (23.7%) |  |
| Type AB | 35 (29.9%) | 21 (25.0%) | 14 (42.4%) |  | 19 (32.8%) | 16 (27.1%) |  |
| Type B1 | 13 (11.1%) | 13 (15.5%) | 0 (0%) |  | 11 (19.0%) | 2 (3.4%) |  |
| Type B2 | 30 (25.6%) | 27 (32.1%) | 3 (9.1%) |  | 17 (29.3%) | 13 (22.0%) |  |
| Type B3 | 12 (10.3%) | 8 (9.5%) | 4 (12.1%) |  | 3 (5.2%) | 9 (15.3%) |  |
| Thymic Carcinoma | 11 (9.4%) | 7 (8.3%) | 4 (12.1%) | 0.005 | 6 (10.3%) | 5 (8.5%) | 0.002 |
| Stage, n (%) |  |  |  |  |  |  |  |
| Stage I | 35 (29.9%) | 26 (31.0%) | 9 (27.3%) | 0.893 | 21 (36.2%) | 14 (23.7%) | 0.625 |
| Stage IIa | 41 (35.0%) | 31 (36.9%) | 10 (30.3%) |  | 18 (31.0%) | 23 (39.0%) |  |
| Stage IIb | 20 (17.1%) | 13 (15.5%) | 7 (21.2%) |  | 9 (15.5%) | 11 (18.6%) |  |
| Stage III | 14 (12.0%) | 9 (10.7%) | 5 (15.2%) |  | 6 (10.3%) | 8 (13.6%) |  |
| Stage IVa | 1 (0.9%) | 1 (1.2%) | 0 (0%) |  | 1 (1.7%) | 0 (0%) |  |
| Stage IVb | 4 (3.4%) | 3 (3.6%) | 1 (3.0%) |  | 2 (3.4%) | 2 (3.4%) |  |
| Missing | 2 (1.7%) | 1 (1.2%) | 1 (3.0%) |  | 1 (1.7%) | 1 (1.7%) |  |

**Table S4**. **Multivariable Cox regression analyses of all-cause mortality in the TCGA-THYM cohort.**

| **All cause mortality** | **HR (95%CI)** | **p-values** |
| --- | --- | --- |
| **Low CDH3** | ref |  |
| **High CDH3** | 26.28(5.41, 127.69) | <0.0001 |
| **Low Risk** | ref |  |
| **High Risk** | 9.22 (1.01, 84.17) | 0.049 |
| **Per 0.1 increase in WSI prediction score** | 1.91 (1.27, 2.88) | <0.01 |

**Adjusted for Age, Sex, Race/ethnicity,** **Pathological classification, Stage**

**Supplementary Figure:**

**Supplementary Figure 1. Integrated single-cell atlas of TETs and identification of malignant epithelial cells.**

(A) UMAP visualization of the integrated single-cell dataset after quality control and batch correction. (B) Dot plot of canonical marker genes used for cell type annotation across the six major cell populations. (C) Stacked bar plot showing the relative proportions of major cell types across individual samples. (D) Feature-density plots showing the spatial enrichment patterns of representative lineage markers across the global cell atlas, including EPCAM, RGS5, PECAM1, MS4A1, and TRBC1. (E) UMAP visualization of reclustered epithelial cells prior to malignant-state identification. (F) UMAP plot of epithelial cells colored by CopyKAT classification, showing separation of aneuploid and diploid populations. (G) Overall and sample-level distributions of CopyKAT-defined groups, including aneuploid, diploid, and unclassified epithelial cells.

**Supplementary Figure 2. Refined annotation of immune and stromal populations and NicheNet analysis of CDH3⁺ malignant epithelial interactions with multiple recipient cell subsets.**

(A) Integrated UMAP visualization of all refined cell subsets across the TET ecosystem. (B) Dot plots showing canonical marker gene expression used to annotate refined T-cell, B-cell, myeloid-cell, and stromal-cell subsets. (C) Stacked bar plot showing the relative abundance of refined cell subsets across individual samples. (D) NicheNet ligand–receptor–target analysis for signaling from C2_CDH3 malignant epithelial cells to M1 macrophages.(E) NicheNet ligand–receptor–target analysis for signaling from C2_CDH3 malignant epithelial cells to fibroblasts.(F) NicheNet ligand–receptor–target analysis for signaling from C2_CDH3 malignant epithelial cells to CD4⁺ naïve T cells.(G) NicheNet ligand–receptor–target analysis for signaling from C2_CDH3 malignant epithelial cells to CD8⁺ naïve T cells.(H) NicheNet ligand–receptor–target analysis for signaling from C2_CDH3 malignant epithelial cells to Treg cells.(I) NicheNet ligand–receptor–target analysis for signaling from C2_CDH3 malignant epithelial cells to naïve B cells.

**Supplementary Figure 3. External spatial transcriptomic validation of the CDH3-associated malignant niche.**

(A) Spatial mapping of the inferred CDH3⁺ using cell2location. (B) Spatial mapping of M1 macrophages. (C) Spatial mapping of M2 macrophages. (D) Spatial mapping of fibroblasts. (E) Spatial feature plots of CDH3 expression. (F) Section-level MISTy analysis. (G) Integrated MISTy analysis.

**Supplementary Figure 4. Derivation of the CDH3-associated bulk transcriptional signature from malignant epithelial single-cell programs.**

(A) Soft-threshold power analysis for hdWGCNA in malignant epithelial cells. (B) Hierarchical clustering dendrogram of genes identified by hdWGCNA. (C) Module membership (kME) distributions for all hdWGCNA-defined gene modules. (D) Dot plot showing module activity across malignant epithelial subpopulations, together with the gene network of the M7 module. (E) Venn diagram showing the overlap between genes assigned to the M7 module and genes upregulated in the C2_CDH3 subpopulation, yielding the 68-gene CDH3-associated signature. (F) Functional enrichment analysis of the 68-gene CDH3-associated signature.

**Supplementary Figure 5. Independent histological, transcriptomic, and multiplex immunofluorescence validation of the CDH3-associated epithelial program in TETs.**

(A) Clinicopathological annotation of the independent in-house TET cohort comprising 32 patients, including age, sex, WHO histological subtype, tumor invasion status, and tumor necrosis status. (B) Representative multiplex immunofluorescence images from the 32 in-house TET samples stained for DAPI, EPCAM, CDH3, and CD45. DAPI, blue; EPCAM, yellow; CDH3, green; CD45, red. (C) HALO-based quantification of total segmented cells across WHO histological subtypes. (D) HALO-based quantification of EPCAM⁺ epithelial-cell fractions among all segmented cells across WHO histological subtypes. (E) HALO-based quantification of CDH3⁺ cell fractions among all segmented cells across WHO histological subtypes. (F) HALO-based quantification of epithelial-normalized CDH3 positivity, calculated as the fraction of EPCAM⁺CDH3⁺ cells among EPCAM⁺ epithelial cells, across WHO histological subtypes. (G) Receiver operating characteristic analysis of the ssGSVA score derived from genes positively associated with the CDH3-high program in TCGA-THYM, used to determine the optimal cutoff for CDH3-low-like and CDH3-high-like classification. (H) Distribution of CDH3-associated ssGSVA scores in TCGA-THYM and the in-house cohort after stratification into CDH3-low/CDH3-high or CDH3-low-like/CDH3-high-like groups. (I) CDH3 expression levels in the in-house cohort stratified by the TCGA-derived ssGSVA cutoff. (J) Distribution of WHO histological subtypes between CDH3-low-like and CDH3-high-like tumors in the in-house cohort. (K) Distribution of tumor invasion status between CDH3-low-like and CDH3-high-like tumors in the in-house cohort. (L) Distribution of tumor necrosis status between CDH3-low-like and CDH3-high-like tumors in the in-house cohort. (M) Comparison of epithelial-normalized CDH3 positivity, calculated as EPCAM⁺CDH3⁺ cells among EPCAM⁺ epithelial cells, between CDH3-low-like and CDH3-high-like tumors within each WHO histological subtype in the in-house cohort. (N) Estimated abundance of the C2_CDH3 malignant epithelial state across WHO histological subtypes and CDH3 groups in TCGA-THYM and the in-house cohort, as inferred by deconvolution analysis. Data are shown as mean ± SEM where applicable, and individual points represent individual tumor samples. P values are shown where indicated.

**Supplementary Figure 6. Genomic, methylation-associated, and transcriptional-regulatory features of CDH3-associated molecular groups and malignant epithelial states in TETs.**

**(A)** Histotype-stratified copy-number alteration profiles generated using GISTIC2.0 for CDH3-low and CDH3-high tumors (CDH3-low = 84; CDH3-high = 33) in the available histological categories, including type A thymoma, type AB thymoma, type B2 thymoma, type B3 thymoma, and thymic carcinoma. Red peaks indicate copy-number gains or amplifications, whereas blue peaks indicate copy-number losses or deletions. Sample numbers for each subgroup are indicated in the corresponding panels. **(B)** inferCNV heatmap showing large-scale inferred copy-number alteration profiles across malignant epithelial subclusters. Rows represent malignant epithelial cells and columns represent genomic positions ordered by chromosome. Red and blue indicate inferred copy-number gains and losses, respectively.**(C)** Distribution of inferred CNV scores across the five malignant epithelial subclusters: C0_PDK4, C1_SLPI, C2_CDH3, C3_GMFG, and C4_RGS1. **(D)** Per-sample SNV counts in the CDH3-low and CDH3-high groups. Each point represents one TCGA-THYM sample. The P value was calculated using a two-sided Wilcoxon rank-sum test. **(E)** Relative proportions of the six major single-base substitution classes in the CDH3-low and CDH3-high groups: C>A, C>G, C>T, T>A, T>C, and T>G.**(F)** Overall mutational-spectrum analysis of CDH3-low and CDH3-high tumors. The upper panels show group-level single-base substitution spectra across trinucleotide contexts. The lower panel shows the estimated exposures of COSMIC SBS signatures in the two molecular groups.**(G)** Histotype-stratified mutational-spectrum analysis. Single-base substitution spectra and COSMIC SBS signature exposures are shown for CDH3-low and CDH3-high tumors within the available histological categories. Sample numbers are indicated in the corresponding panels.**(H)** Global DNA methylation levels in TCGA-THYM tumors. **(I)** Heatmap of the top 200 differentially methylated probes between CDH3-low and CDH3-high tumors. **(J)** Functional enrichment analysis of genes associated with differentially methylated probes. **(K)** Binarized SCENIC regulon activity heatmap across malignant epithelial subclusters. Rows represent selected cluster-associated TF regulons and columns represent individual malignant epithelial cells. Regulons were assigned to the subcluster in which they showed the highest regulon specificity score. Blue indicates an active regulon state (ON), whereas gray indicates an inactive state (OFF).

**Supplementary Figure 7. Immune and stromal differences between CDH3-low and CDH3-high TET subgroups.**

(A) IOBR-based comparison of immune and stromal signature scores between CDH3-low and CDH3-high tumors. (B) Heatmap showing differential enrichment of representative immune, CAF, TGF-β, EMT, and macrophage-related programs across the two groups. (C–E) Quantitative comparisons of M2 macrophage, CAF, and EMT scores between CDH3-low and CDH3-high tumors. (F–G) Representative histopathological images of CDH3-low and CDH3-high tumors, respectively. ***P < 0.001 versus the Group Low.

**Supplementary Figure 8. Thymic carcinoma organoid establishment and marker validation.**

(A) Organoid morphology and CDH3 expression in thymic carcinoma organoids. (B) Marker staining confirming thymic carcinoma identity of the organoids.

**Supplementary Figure 9. CDH3-specific knockdown suppresses thymic carcinoma xenograft growth in vivo.**

(A) Schematic overview of the xenograft experiment using ThyL6 cells stably transduced with control or CDH3-targeting lentiviral shRNA. A total of 1 × 10⁶ ThyL6 cells were subcutaneously injected into nude mice, and tumor volume was measured every 3 days until tumor collection (n = 7 mice per group). (B) Validation of lentiviral transduction efficiency and CDH3 knockdown in ThyL6 cells. Representative fluorescence images show successful lentiviral transduction, and qRT-PCR confirms reduced CDH3 expression in sh-CDH3 cells compared with control cells. (C) Representative gross images of excised xenograft tumors and tumor-bearing mice from the Sham and sh-CDH3 groups at endpoint. (D) Tumor growth curves showing longitudinal changes in tumor volume in the Sham and sh-CDH3 groups. (E) Body weight curves of mice in the Sham and sh-CDH3 groups during the xenograft experiment. (F) Comparison of tumor weight between the Sham and sh-CDH3 groups at endpoint. (G) Comparison of tumor index, calculated as tumor weight divided by body weight, between the Sham and sh-CDH3 groups at endpoint. (H) Representative hematoxylin and eosin staining and immunohistochemical staining of xenograft tumors from the Sham and sh-CDH3 groups, including CD5 and CDH3 staining. Data are shown as mean ± SEM. **P < 0.01 versus the Group Sham.

**Supplementary Figure 10. Therapeutic vulnerability analyses associated with the CDH3-high malignant program in TETs.**

(A) Comparison of TIDE scores between the CDH3-low and CDH3-high groups in the overall TCGA-THYM cohort (n = 117; CDH3-low = 84; CDH3-high = 33) and within the available histological categories, including type A thymoma, type AB thymoma, type B2 thymoma, type B3 thymoma, and thymic carcinoma. (B) InstaPrism-based estimation of malignant epithelial subpopulation abundance in the independent pembrolizumab-treated advanced thymic carcinoma cohort (GSE181815, n = 10, responders = 5, non-responders = 5), comparing responder (R) and non-responder (NR) groups. (C) BeyondCell analysis of drug sensitivity in epithelial tumor cells. (D) Correlation analysis between CDH3 expression and predicted drug sensitivity in the TCGA cohort using oncoPredict. (E) Overlap of candidate compounds identified from single-cell and bulk-level drug sensitivity analyses. (F) Molecular docking of BMS-754807 and MK-2206 to CDH3. (G) Covariance matrix and Gibbs free energy landscape analysis of the MK-2206–CDH3 complex from molecular dynamics simulation. (H) Root mean square deviation (RMSD) plot of the MK-2206–CDH3 complex. (I) Radius of gyration plot of the MK-2206–CDH3 complex. (J) Hydrogen bond profile of the MK-2206–CDH3 complex. (K) Solvent accessible surface area (SASA) profile of the MK-2206–CDH3 complex.

**Supplementary Figure 11. Additional in vivo and transcriptomic characterization of drug-treated xenograft tumors.**

(A) Representative gross images of mice at endpoint. (B) PCA of xenograft RNA-seq data (n = 3 tumors per group). (C) Differential expression and pathway enrichment analysis for BMS-754807 versus Sham. (D) Differential expression and pathway enrichment analysis for MK-2206 versus Sham.

**Supplementary Figure 12. Representative high- and low-risk cases illustrate distinct pathology attention patterns and tumor–macrophage spatial relationships.**

(A) Representative high-risk TCGA case showing the original H&E image, tissue segmentation result, and model-derived attention heatmap. (B) Representative low-risk TCGA case showing the original H&E image, tissue segmentation result, and model-derived attention heatmap.
